# Identification and characterization of a set of conserved and new regulators of cytoskeletal organization, cell morphology and migration

**DOI:** 10.1186/1741-7007-9-54

**Published:** 2011-08-11

**Authors:** Siau Wei Bai, Maria Teresa Herrera-Abreu, Jennifer L Rohn, Victor Racine, Virginia Tajadura, Narendra Suryavanshi, Stephanie Bechtel, Stefan Wiemann, Buzz Baum, Anne J Ridley

**Affiliations:** 1 Randall Division of Cell and Molecular Biophysics, King's College London, New Hunt's House, Guy's Campus, London SE1 1UL, UK; 2 MRC Laboratory of Molecular Cell Biology, University College London, Gower Street, London WC1E 6BT, UK; 3 Institute of Molecular and Cellular Biology, 61 Biopolis Drive, Proteos, 138673, Singapore; 4 Deutsches Krebsforschungszentrum, Im Neuenheimer Feld 280, Heidelberg, Germany; 5 SWB, Institut Pasteur, 25-28 rue du Docteur Roux, 75015 PARIS; VR, Fluofarma, 2 rue Robert Escarpit, 33600 Pessac, France

## Abstract

**Background:**

Cell migration is essential during development and in human disease progression including cancer. Most cell migration studies concentrate on known or predicted components of migration pathways.

**Results:**

Here we use data from a genome-wide RNAi morphology screen in *Drosophila melanogaster *cells together with bioinformatics to identify 26 new regulators of morphology and cytoskeletal organization in human cells. These include genes previously implicated in a wide range of functions, from mental retardation, Down syndrome and Huntington's disease to RNA and DNA-binding genes. We classify these genes into seven groups according to phenotype and identify those that affect cell migration. We further characterize a subset of seven genes, *FAM40A, FAM40B, ARC, FMNL3, FNBP3/FBP11, LIMD1 *and *ZRANB1*, each of which has a different effect on cell shape, actin filament distribution and cell migration. Interestingly, in several instances closely related isoforms with a single *Drosophila *homologue have distinct phenotypes. For example, *FAM40B *depletion induces cell elongation and tail retraction defects, whereas *FAM40A *depletion reduces cell spreading.

**Conclusions:**

Our results identify multiple regulators of cell migration and cytoskeletal signalling that are highly conserved between *Drosophila *and humans, and show that closely related paralogues can have very different functions in these processes.

## Background

Cell migration involves the coordinated regulation of cytoskeletal dynamics and cell adhesion turnover, and is directed by extracellular stimuli, including chemokines, cytokines, growth factors and the extracellular matrix [1,2]. Cell migration is essential for embryonic development and wound healing, but also contributes to the pathogenesis of human diseases, such as cancer, autoimmune diseases and chronic inflammation. Targeted inhibition of molecules involved in cell migration could, therefore, be used to treat several human diseases. Many intracellular signalling proteins have been implicated in cell migration, and, in particular, Rho GTPases are known to contribute to multiple cellular processes that affect cell migration [3]. Regulators of actin cytoskeletal dynamics, including formins and WASP/WAVE-related proteins, are key targets of cell migration signalling [4].

The contribution of proteins to cell migration can be assessed using scratch wound assays, which quantify the time required for cells to migrate into and fill a gap created in a cell monolayer. Several groups have recently adapted this type of approach for high-throughput RNAi screening to identify new regulators of cell migration. An RNAi screen for scratch-wound closure in MCF10A breast epithelial cells that targeted all kinases and phosphatases, together with other selected genes implicated in cell migration, revealed that cell speed in this model was increased by knockdown of genes that reduced cell-cell adhesion [5]. Using a similar approach, a screen of siRNAs targeting over 5,000 genes in SKOV3 ovarian cancer cells identified five genes including three kinases that potently reduced cell migration [6]. Other screening approaches have also identified novel regulators of cell migration. For example, genome-wide RNAi screen in *Caenorhabditis elegans *identified 99 genes that affected migration of the distal tip cells during gonadogenesis [7].

Since many of the genes identified in these screens affect cell migration indirectly, for example, by altering cell division and growth, we chose to take a different approach. To select for potential novel regulators of cell migration we used a morphology screen in *Drosophila *cells to identify conserved proteins that alter cell shape and actin filament distribution. RNAi was then used to test the roles of the human counterparts of these genes in cytoskeletal organisation and cell migration. This approach proved highly effective in identifying functionally conserved genes, identifying 26 conserved human proteins that are required for normal cytoskeletal organisation and cell morphology in prostate cancer-derived PC3 cells and HeLa cells. Significantly, a large number of these putative novel cytoskeletal regulators were found to alter cell migration and several have previously been implicated in human diseases, providing a new set of potential therapeutic targets.

## Results

### Selection of putative motility modifier genes

We used the results of a genome-wide RNAi morphological screen in *Drosophila melanogaster *S2R+ cells [8] to select genes that altered the shape of S2R+ cells, but for which the function in *Drosophila *was not previously known. A bioinformatic approach was then used to select a subset of 16 of these genes for which there was little or no evidence of a role in regulating cell shape, but for which there was some other information on their function (Additional file 1, Table S1). We used functional information from studies of their homologues in other organisms (yeast, *C. elegans*, mammals), on whether they were hits in any other genetic screens, had known interaction partners in humans or other species (for example, *Saccharomyces cerevisiae*) or putative interaction partners based on large scale screens, and known or predicted protein domains (Table S1; see Materials and methods). For example, CG12505 was selected because the mammalian homologue ARC is known to bind to actin, whereas CG3542 was selected because a human homologue HYPC interacts with Huntingtin. The human homologues of these 16 genes were identified (Table 1), yielding a group of 26 human genes. Nine of the *Drosophila *genes have a single human homologue, while the remaining genes have two or three human paralogues. These genes were named 'putative motility modifiers' (PMMs).

**Table 1:** List of human PMMs

*Drosophila *gene	Human gene symbol	Human name	Alternative names
**CG12505**	ARC	activity-regulated cytoskeleton-associated protein	Arg3.1, KIAA0278
**CG31132**	BRWD1	bromodomain and WD repeat domain containing 1	N143, WDR9, FLJ43918, C21orf107
	BRWD3	bromodomain and WD repeat domain containing 3	BRODL, MRX93, FLJ38568
	PHIP	pleckstrin homology domain interacting protein	WDR11, FLJ20705, ndrp, FLJ45918, MGC90216
**CG10671**	C20orf142	chromosome 20 open reading frame 142	FITM2. MGC30135, dJ881L22.2
**CG14995**	C21orf2	chromosome 21 open reading frame 2	YF5, A2
**CG33130**	CAMSAP1	Calmodulin-regulated spectrin-associated protein 1	PRO2405, FLJ31228, MGC163452, bA100C15.1
**CG11339**	EPB41L4A	erythrocyte membrane protein band 4.1 like 4A	NBL4, FLJ38738
**CG11526**	FAM40A	family with sequence similarity 40, member A	FLJ14743, MGC148091, RP4-773N10.1, STRIP1
	FAM40B	family with sequence similarity 40, member B	STRIP2
**CG32138**	FMNL1	formin-like 1	FMNL, FHOD4, FRL1, KW-13, C17orf1, C17orf1b, MGC1894
	FMNL2	formin-like 2	FHOD2, FLJ37546
	FMNL3	formin-like 3	WBP3, FHOD3, FRL2, FLJ45265, MGC45819, DKFZp762B245
**CG3542**	FNBP3	formin-binding protein 3; pre-mRNA processing factor 40 homolog A	PRPF40A, HYPA, FLJ20585, FBP11, NY-REN-6, HIP10, FLAF1
	HYPC	Huntingtin interacting protein C, pre-mRNA processing factor 40 homolog B	PRPF40B
**CG11505**	LARP4	La -related protein 4	PP13296, C-Mpl binding protein, MGC74631
**CG11063**	LIMD1	LIM domain containing 1	None
	WTIP	Wilms tumor 1 interacting protein	None
**CG10362**	PDZK8	PDZ domain containing 8	PDZD8, bA129M16.2, FLJ34427, FLJ25412
**CG31012**	SH3D19	SH3 domain protein D19	EBP, EVE1, Kryn, SH3P19
	SH3KBP1	SH3-domain kinase binding protein 1	CIN85, HSB-1, GIG10, MIG18, CD2BP3
**CG5965**	ZMYM3	zinc finger, MYM-type 3	ZNF261, ZNF198L2, XFIM, DXS6673E, KIAA0385
	ZMYM4	zinc finger, MYM-type 4	ZNF262, KIAA0425, ZNF198L3, CDIR
	ZMYM6	zinc finger, MYM-type 6	ZNF258, ZNF198L4
**CG12299**	ZNF135	zinc finger protein 135	pT3, ZNF61, pHZ-17, ZNF78L1
**CG9448**	ZRANB1	zinc finger, RAN-binding domain containing 1	TRABID, DKFZp762P2216

The 16 *Drosophila *gene names and the HGNC gene symbol (human gene symbol) and full name (human name) of the 26 human homologues are shown. Alternative names (taken from NCBI Gene) are listed.

### Morphology screen of PMM genes

**Figure 1:**
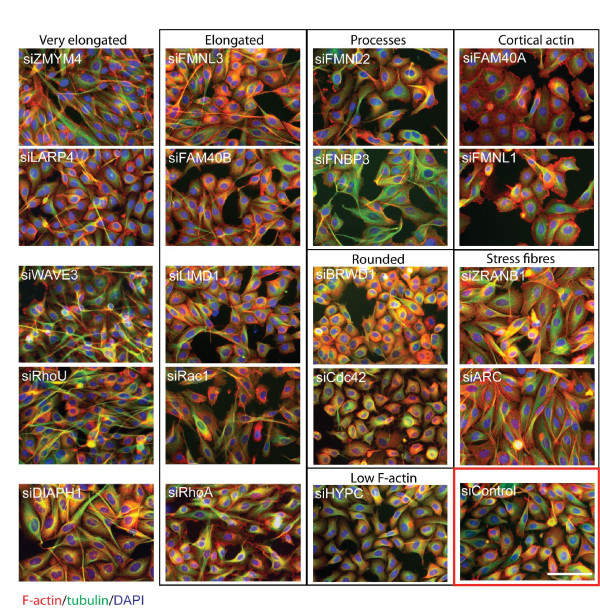
**Effects of PMM depletion on cell morphology**. PC3 cells were transfected with the indicated siRNA pools for each PMM or for known regulators of actin dynamics in 384-well plates. After 72 h, cells were fixed and stained for F-actin (red), tubulin (green) and nuclei (blue). Cells are divided into groups based on their morphology. Scale bar, 100 μm.

To identify roles for these genes in regulating cell morphology in human cells, each of the 26 human PMM genes was knocked down in PC3 prostate carcinoma cells, which grow and migrate as single cells without strong cell-cell adhesions, using pools of four siRNAs. In this analysis, 25 of the 26 PMMs had a discernable effect on cytoskeletal organization and/or cell shape (Figure 1; Table 2; Additional file 2, Figure S1). In order to identify genes that induced similar changes and, hence, could act on a similar pathway, cells were classified for 14 different parameters: 7 distinct F-actin distributions, presence of microtubule-containing processes, 3 different cell shapes, spread area, and the presence of multinucleate cells (Table 2, examples of phenotypes are shown in Figure 1 and Additional file 3, Figure S2). These were compared to the phenotypes induced by knockdown of known regulators of the actin cytoskeleton, including the Rho GTPases RhoA, Rac1, Cdc42 and RhoU/Wrch1 and the actin polymerization regulators CAPZB, cofilin (CFL1), mDia1 (DIAPH1) and WAVE3.

**Table 2:** Cell morphology and cytoskeleton organisation in PMM-depleted PC3 cells.

Group	Gene symbol	Actin	Microtubules	Cell shape	Spread area	Multi-nucleate
	**control**	**stress fibres 4%** **strong patches 8%** **cortical actin 12%** **low 14%**	**processes 6%**	**elongated 10%** **rounded 4%**	**larger 4%**	**0%**
**1**	ARC	**stress fibres 70%**	NP	elongated	larger	6%
	ZRANB1	**stress fibres 90%**	NP	elongated	larger	10%
**2**	C20orf142	cortical actin	NP	NP	**larger 85%**	NP
	CAMSAP1	cortical actin, spots	NP	NP	**larger 85%**	NP
	FMNL1	**cortical actin 90%**, rough edge	NP	NP	larger	NP
	FAM40A	**cortical actin 90%**, rough edge	NP	NP	NP	NP
	C21orf2	**cortical actin 55%**	NP	NP	NP	NP
**3**	FMNL2	cortical actin, spots	**processes 60%**	NP	larger	9%
	ZNF135	spots	**processes 50%**	NP	NP	NP
	ZMYM3	cortical actin, spots	**processes 25%**	NP	larger	NP
	PHIP	cortical actin	**processes 35%**	NP	NP	NP
**4**	FAM40B	cortical actin	processes	**elongated 65%**	NP	NP
	FNBP3	cortical actin, stress fibres	processes	**elongated 65%**	larger	4%
	FMNL3	NP	processes	**elongated 80%**	larger	NP
	*Rac1*	stress fibres	NP	**elongated 65%**	larger	NP
	LIMD1	NP	processes	**elongated 50%**	NP	NP
	SH3KBP1	NP	NP	**elongated 90%**	NP	NP
	*RhoA*	strong patches	NP	**elongated 70%**	NP	NP
	SH3D19	spots	NP	**very elongated 90%**	NP	NP
**5**	ZMYM4	spots	processes	**very elongated 95%**	NP	NP
	*WAVE3*	spots	processes	**very elongated 90%**	NP	NP
	LARP4	NP	processes	**very elongated 45%**	NP	NP
	*DIAPH1*	NP	processes	**very elongated 90%**	larger	NP
	*RhoU*	NP	NP	**very elongated 80%**	NP	NP
**6**	*CAPZB*	**strong patches 90%**, cortical actin	NP	NP	NP	NP
	*CFL1*	**strong patches 80%**	NP	NP	NP	31%
	PDZK8	**strong patches 45%**	NP	NP	NP	2%
**7**	ZMYM6	rough edge	NP	**rounded 65%**	NP	NP
	*Cdc42*	rough edge	processes	**rounded 70%**	NP	NP
	WTIP	NP	NP	**rounded 95%**	NP	NP
	BRWD1	NP	NP	**rounded 80%**	smaller	NP
	BRWD3	NP	NP	**rounded 80%**	smaller	NP
	HYPC	**low 65%**, smooth	NP	NP	NP	NP
	EPB41L4A	NP	NP	NP	NP	NP

**Figure 2:**
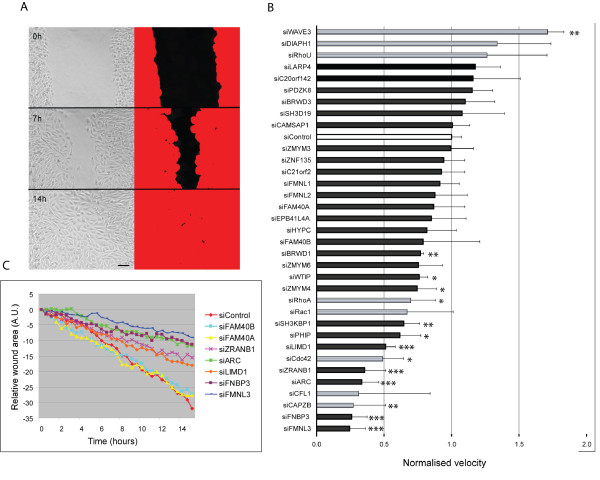
**Effects of PMMs on migration into scratch wounds**. PC3 cells were transfected with siRNA pools for each PMM in 96-well plates. After 72 h, cells were scratch wounded and then wound closure monitored by time-lapse microscopy. **(A) **Examples of images from control siRNA-transfected cells. Phase contrast images (left panels). To quantify wound closure, phase-contrast images were thresholded (right panels). **(B) **Example graph of wound closure for seven PMMs and control. Using thresholded images, the relative wound area is determined at each time point. A.U., arbitrary units. **(**C)**** Relative velocity of cells migrating into wound, normalized to control. PMMs are shown as black bars; known regulators of actin dynamics as grey bars. Those above control siRNA (white bar) stimulated migration; those below inhibited migration. Results show the mean +/- s.d. of four to eight wound areas from two experiments; * *P *≤ 0.05, ** *P *≤ 0.01, ***P ≤ 0.001 compared to control, two-tailed unpaired t-test.

Phenotypes were classified three days after transfection with siRNA pools targeting each of the 26 PMMs or 8 known regulators of the actin cytoskeleton (*italics*). Cells were fixed and stained for F-actin, microtubules, and DNA (as in Figure 1). Three to four different images from two different screens were analysed; total number of cells analysed/condition ≥ 68. The most prominent phenotype for each gene is highlighted in bold text and the percent of cells showing this phenotype is shown in brackets (see Figure 1, Supplementary. Figure 2 for examples of each phenotype). Definitions of actin phenotypes: stress fibres, multiple thick straight F-actin cables/cell; cortical actin, increased F-actin on regions of the plasma membrane; spots, small bright regions of F-actin; strong patches, large bright regions of F-actin; rough edge, free edges of cells are wavy or indented; smooth, F-actin levels similar throughout cytoplasm; low, lower F-actin levels than control cells. Microtubule phenotype: processes, long narrow membrane extensions containing microtubules. Definitions of cell shape: larger, spread area increased compared to control cells; elongated, cell body length ≥ 2 × width; very elongated, cell body length ≥ 3 × width; rounded, length ~ = width. NP, no phenotype.

This analysis of parameters revealed seven major groups of genes based on their predominant phenotype in the siRNA screen (Table 2), which could reflect functional interaction between members of each group. For comparison, the percent of control cells demonstrating each parameter is shown.

The first PMM group, *ARC *and *ZRANB1 *(also known as *TRABID *[9]), both induced a strong increase in stress fibres, which are normally only rarely observed in PC3 cells (Figure 1; Additional file 3, Figure S2). They also increased the spread area and the percent of multinucleate cells (Table 2). ZRANB1/TRABID is a deubiquitinase previously linked to Wnt signalling [9], whereas ARC binds to actin filaments [10]. The similar phenotype induced by these two genes suggests that ZRANB1 might regulate ARC. Of the known actin regulators, only *Rac1 *depletion increased stress fibres, although to a lesser extent than *ZRANB1 *or *ARC*.

PMM group 2 increased the level of cortical actin filaments (Additional file 3, Figure S2) and often altered the membrane morphology. This included *FAM40A *and *FMNL1*, which showed an increase in F-actin-rich lamellae around the cell periphery. A subset also made the cells larger, presumably by promoting cell spreading through the cortical F-actin changes. PMM group 3 also affected cortical F-actin but were most striking for the presence of long thin microtubule-containing processes as their most dominant phenotype. The cell shape was not altered, but at least 25% of the cells had one narrow straight process that often extended over multiple neighbouring cells (Additional file 3, Figure S2). Occasionally cells had two processes. Note that depletion of several PMMs in groups 4 and 5 also induced an increase in cells with microtubule-containing processes (Table 2). This suggests that all these proteins are linked in normally suppressing microtubule-based protrusions. This could reflect the known role of the actin cytoskeleton in limiting the generation of microtubule-based protrusions as previously described in *Drosophila *cells in culture [11]. Knockdown of PMM group 4 genes, including *FAM40B *and *FMNL2*, induced an elongated phenotype (Additional file 3, Figure S2). Group 5 genes were characterized by a very elongated bipolar phenotype (Additional file 3, Figure S2), and included *RhoU *and the formin *mDia1 (DIAPH1*). RhoU has previously been reported to affect integrin-mediated adhesions [12], and thus this very elongated phenotype could reflect altered adhesion, and/or changes to localized actin polymerization. Notably, WAVE3, an Arp2/3 complex actin nucleation promoting factor that is known to contribute to lamellipodium formation, was also in this group [13]. PMM group 6 genes had strong patches of F-actin, and included genes encoding the actin filament capping protein CAPZB, the actin filament severing and depolymerising protein Cofilin1 (CFL1), and PDZK8. CFL1 and CAPZB depletion are known to reduce actin filament depolymerization and capping respectively, thereby promoting focal increases in F-actin [14]. Very little is known about PDZK8 except that it interacts with HIV Gag protein and promotes HIV infection. Interestingly, cofilin plays a role in HIV entry through the HIV receptor CXCR4 [15]. Our data suggest that PDZK8 and CFL1 could act together during this process. PMM group 7, including *Cdc42*, induced a rounded cell shape as the predominant phenotype (Additional file 2, Figure S1), likely reflecting a decrease in cell spreading [16]. This includes the closely related homologues *BRWD1 *and BRWD3, which are likely to encode transcriptional regulators (Additional file 1, Table S1), and, hence, might regulate the expression of other genes in the group.

*HYPC *was unique in leading to a reduction in F-actin levels (Figure 1; Additional file 2, Figure S1), and did not fit into any of the groups. Finally, *EPB41L4A *did not induce any discernable phenotype in PC3 cells, although it was selected on the basis of its morphological phenotype in *Drosophila *S2R+ cells. It is possible that *EPB41L4A *is not expressed in PC3 cells.

Interestingly, closely related homologues were generally in different groups; for example, the formins *FMNL1, FMNL2 *and *FMNL3*, and *FAM40A *and *FAM40B *(Table 2).

### Cell migration screen of PMM genes

To determine whether the morphological changes induced by PMM depletion led to changes in migration, we tested the effects of knocking down expression of each PMM on cell migration using scratch wound assays. Migration of cells was monitored by time-lapse microscopy (Figure 2A), and the change in wound size over time was quantified using an automated method of image analysis (Figure 2B). These results were used to generate graphs showing the relative velocity of wound closure (Figure 2C). Of the PMMs, depletion of *FMNL3, FNBP3, ARC, ZRANB1 *and *LIMD1 *led to the strongest inhibition of wound closure. Notably, *ARC *and *ZRANB1 *form PMM group 1, and have an increase in stress fibres and larger spread area (Table 2). Stress fibres can often inhibit cell migration [17]. *FMNL3, FNBP3 *and *LIMD1 *are all part of PMM group 4, and have an elongated shape. Interestingly, the other members of this group also reduced migration (*SH3KBP1, RhoA, Rac1, FAM40B*). By contrast, closely related homologues of *FMNL3 (FMNL1 *and *FMNL2*) and *FNBP3 *(*HYPC*) were in different groups and did not significantly affect migration (Figure 2B). Opposite to group 4 PMMs, the group 5 PMMs *WAVE3, DIAPH1, RhoU *and *LARP4 *(very elongated cells) tended to increase rather than decrease cell migration (Figure 2). Taken together, these results imply that either an increase in stress fibres or an elongated phenotype inhibits migration of PC3 cells into scratch wounds, whereas very elongated bipolar cells can migrate more rapidly.

### Selection of subset of seven PMMs

From the 26 PMMs, a subset of 7 was chosen for further analysis, based on their phenotypes in the morphology and migration screens. These genes included the five that most strongly inhibited migration in scratch wounds when knocked down, *ARC, ZRANB1, FMNL3, FNBP3 *(also known as *FBP11/HYPA*) and *LIMD1 *(Figure 2B, C). We also included two closely related genes, *FAM40A *and *FAM40B*, which had only a small effect on migration in scratch wound assays (Figure 2B, C), but which each had profound effects on cell shape (Figure 1): *FAM40A *depletion resulted in flatter cells, whereas *FAM40B *depletion increased elongation and is in the same PMM group as *FMNL3, FNBP3 *and *LIMD1*. To validate these hits, we began by deconvoluting each siRNA pool to test the phenotypic effects of using the four single siRNAs individually. At least three of the four single oligonucleotide siRNAs induced a similar phenotype to that seen using the pool in PC3 cells and/or HeLa cells (Additional file 4, Table S2; for HeLa cells, see Figure 6), implying that the henotypes arise as the result of RNAi-mediated silencing of the targeted gene rather than off-target effects.

**Figure 3:**
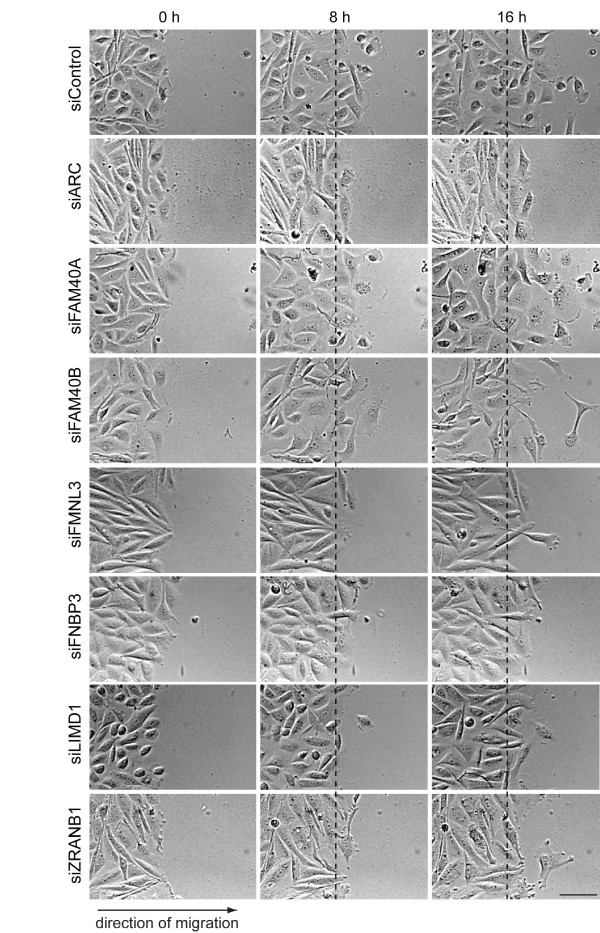
**Effects of PMM depletion on scratch wound healing**. Time series (0, 8 and 16 h after wounding) showing the progression of wound healing in PMM-depleted PC3 cells. Scale bar, 100 μm.

To analyse further how the seven selected PMMs affect cell migration, we imaged cells migrating into the wound. Most control siRNA-transfected cells detached from their neighbours and migrated directionally to fill the gap exposed as the result of the scratch wound (Figure 3, Additional file 5, Movie 1). As expected from the quantification of scratch wound migration (Figure 2C), *ARC, ZRANB1, FMNL3, FNBP3 *and *LIMD1 *depletion strongly inhibited migration into the wound (Figure 3; Additional file 6, Movie 2; Additional file 7, Movie 3; Additional file 8, Movie 4; Additional file 9, Movie 5, Additional file 10, Movie 6). Most cells did not detach from neighbouring cells, in contrast to the control siRNA-transfected cells. *FAM40B *is in the same PMM group 4 as *FMNL3, FNBP3 *and *LIMD1*, but did not affect scratch wound migration as strongly (Figure 2C). Depletion of each of these PMM group 4 genes induced an elongated phenotype in the movies, but *FAM40B*-depleted cells were able to detach from neighbours and could move further into the wound (Figure 3; Additional file 11, Movie 7), *FAM40A*-depleted cells were also able to migrate into the wound but migrated with a very different phenotype to *FAM40B*-depleted cells. They had broad lamellipodia extending into the wound edge, consistent with the broad lamellar phenotype observed in the morphology screen (Figure 3; Additional file 12, Movie 8).

### Effects of selected PMMs on PC3 cell cytoskeletal organization and migration

**Figure 4:**
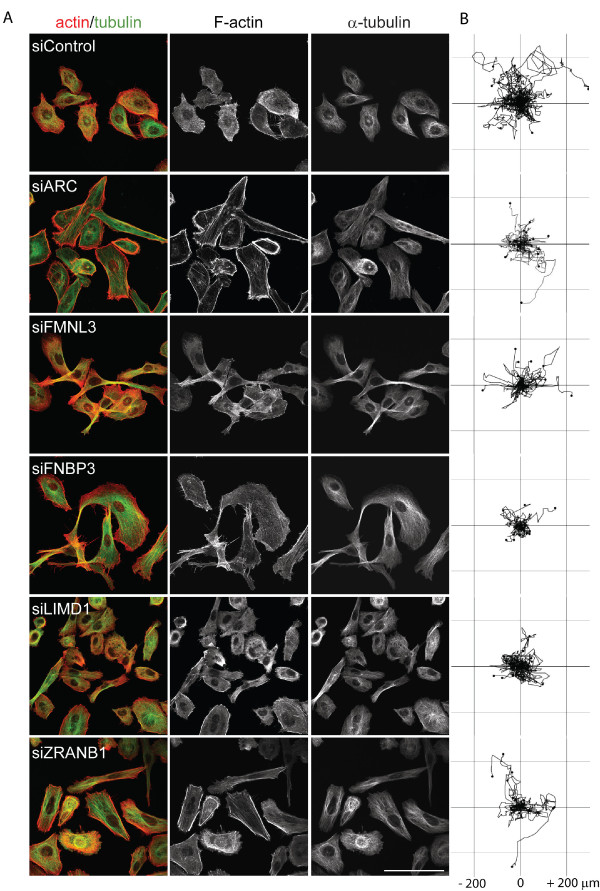
**Selected PMMs have different effects on the actin cytoskeleton and migration**. PC3 cells were transfected with the indicated siRNA pools for each PMM. **(A) **After 24 h, cells were seeded at subconfluence on coverslips, then fixed after 48 h and stained for F-actin (red) and α-tubulin (green). Scale bar, 100 μm. **(B) **Cells were seeded on plastic dishes and monitored by time-lapse microscopy for 14 h. Cells were tracked using ImageJ software (Chemotaxis and Migration Tool plugin). Tracks for 25 cells randomly selected from 70 to 99 cell tracks from three different movies for each PMM are shown.

**Figure 5:**
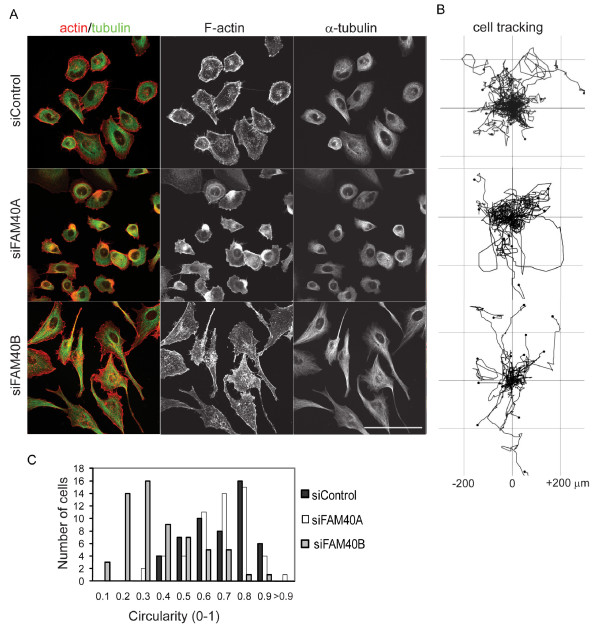
**FAM40A and FAM40B have opposite effects on cell shape and migration**. PC3 cells were transfected with the siRNA pools targeting *FAM40A*, *FAM40B *or control siRNA. **(A) **After 24 h, cells were seeded at subconfluence on coverslips, then fixed after 48 h and stained for F-actin (red) and tubulin (green). **(B) **Cells were seeded on plastic dishes and monitored by time-lapse microscopy for 14 h. Cells were tracked using ImageJ software (Chemotaxis and Migration Tool plugin). Tracks for 25 cells randomly selected from 70 to 99 cell tracks from three different movies for each of control, FAM40A and FAM40B are shown. **(C) **The circularity of cells in (A) was determined using ImageJ (Circularity plugin). A circularity value of 1.0 indicates a perfect circle. As the value approaches 0, it indicates an increasingly elongated polygon.

The seven selected PMMs were further analysed for their effects on morphology and migration of sub-confluent PC3 cells (Figures 4 and 5). PC3 cells normally have very few stress fibres, but *ARC *and *ZRANB1 *depletion led to the formation of stress fibres and strong cortical F-actin bundles at the periphery of cells (Figure 4), as already observed in the screen analysis (Figure 1, Table 2). *LIMD1*-depleted cells also often exhibited strong cortical F-actin bundles but no stress fibres, and in many cells microtubules were disorganized without any clear association with an MTOC. *FMNL3 *and *FNBP1 *knockdown cells were elongated and had long protrusions that extended over neighbouring cells, often with multiple short actin filament bundles at their tips. Microtubules extended right along these protrusions into the tips. *FAM40B*-depleted cells also often had very long thin protrusions containing microtubules, but showed no change in F-actin bundling compared to control cells (Figure 5A), and thus were clearly distinct from *FMNL3 *and *FNBP1 *knockdown cells. This correlates with the scratch wound analysis, which indicated that the *FAM40B *morphology was different from *FMNL3 *or *FNBP1 *(Figure 3). *FAM40B*-depleted cells were more elongated than control cells (Figure 5A), as already observed from the screen analysis (Table 2). *FAM40A *knockdown cells were less spread than controls and often had a broad lamellipodium (Figure 5A, B). Some cells had an intense region of F-actin staining at the opposite side to the lamellipodium, resembling the uropod of leukocytes [18].

To determine the effect of each PMM on the random migration of single PC3 cells, time-lapse movies of PMM-depleted cells were used to generate cell tracks (Figures 4 and 5, right panels) and determine cell migration speed (Additional file 13, Figure S3). *FNBP3*-depleted cells showed the least displacement from their point of origin and lowest migration speed, and *LIMD1, ARC, FMNL3 *and *ZRANB1 *knockdown cells all showed a strong decrease in migration speed, correlating with their migratory behaviour in the scratch wound assay (Figure 2). Only *FAM40A*-depleted cells had a similar migration speed to control cells (Additional file 13, Figure S3).

### Phenotypes of PMM-depleted HeLa cells

**Figure 6:**
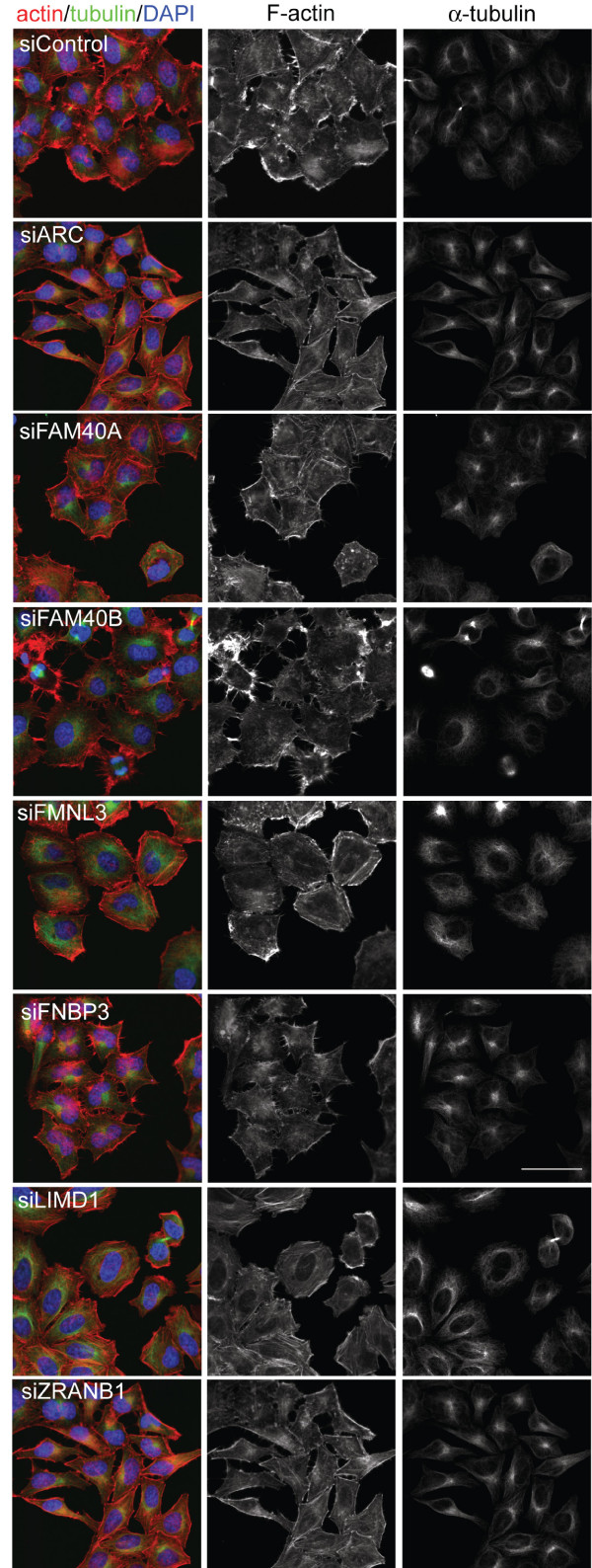
**Effects of PMM depletion on cytoskeletal organisation in HeLa cells**. HeLa cells were transfected with the siRNA pools for the indicated PMMs in thin-bottomed multi-well plates then fixed after 72 h and stained for F-actin (red), tubulin (green) and DNA (DAPI, blue). Images shown are the maximum projection of a z-stack; scale bar, 50 μm.

To determine whether depletion of each of the seven selected PMMs induced a similar phenotype in a different cell type, we tested their effects in HeLa cells (Figure 6). *ARC, LIMD1 *and *ZRANB1 *depletion had qualitatively similar effects in HeLa cells to PC3 cells. *ARC *depletion led to a strong increase in thick actin filament bundles at the edges of cells, and in some cells to an increase in stress fibres, similar to the response of PC3 cells. *LIMD1 *depletion increased stress fibres and/or cortical F-actin bundles in a proportion of cells, somewhat similar to ARC depletion. *ZRANB1 *knockdown led to a strong increase in F-actin bundles along cell edges and some increase in stress fibres, although these were not as thick as those in ARC or LIMD-depleted cells. *ZRANB1 *knockdown cells also had fewer lamellipodia.

**Figure 7:**
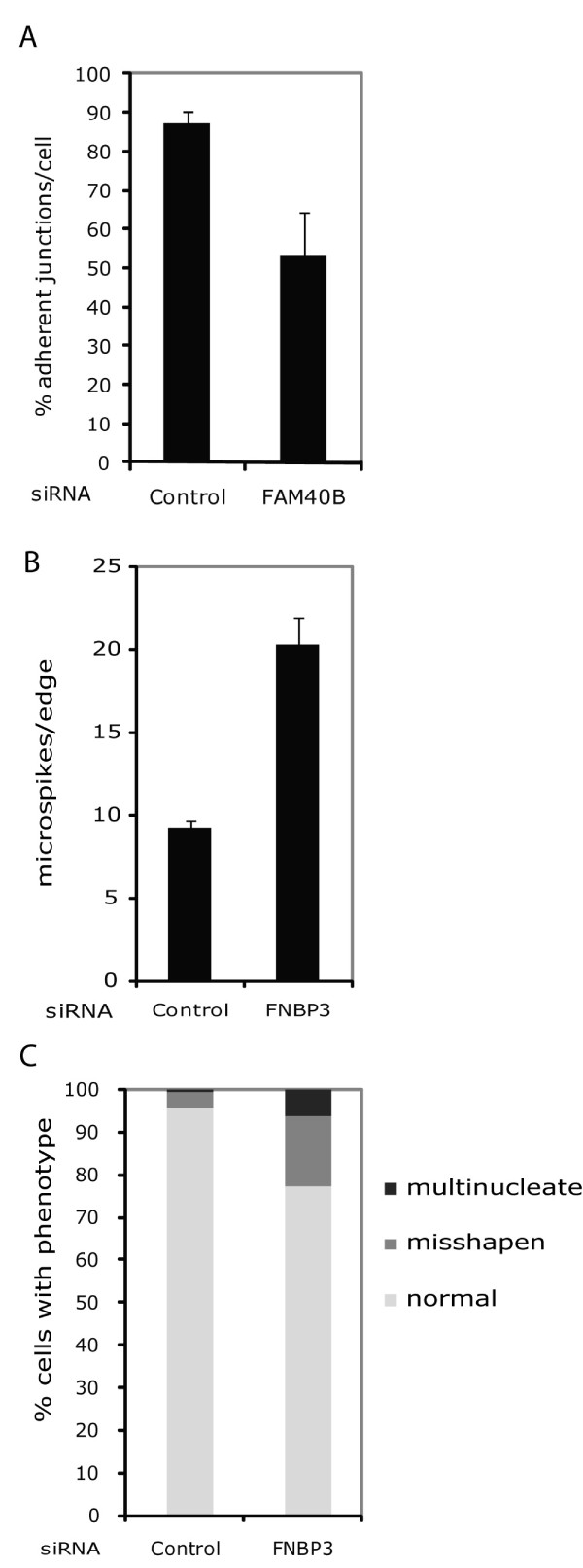
**Quantification of PMM depletion phenotypes in HeLa cells**. Images from HeLa cells as described in Figure 6 were quantified for phenotypical changes. **(**A**)** Cell-cell adhesion. A junction was counted as adherent if more 50% of the length of that cell side was in contact with the neighbouring. The number of sides with junctions was divided by the total number of sides to give the percentage of adherent junctions. The number of sides varied between three to eight/cell. In each experiment 40 cells in each of two images were quantified per condition; therefore, total cell number analysed/condition = 120. **(B)** Microspikes. In each experiment, protrusions from the cell edge were counted for 16 cells in each of two images; therefore, the total number of cell edges analysed/condition = 96. **(**C**)** Nuclear morphology. In each experiment, three fields of cells (50 to 200 cells/field) were counted per condition. Only interphase cells were counted. Misshapen nuclei were defined as those deviating substantially from an oval shape. Graphs show the mean ± SD of two independent experiments.

The effects of *FAM40A, FAM40B, FMNL3 *and *FNBP3 *depletion were different between the two cell types, probably reflecting the different intrinsic phenotypes of HeLa versus PC3 cells. In contrast to PC3 cells, HeLa cells exhibit cell-cell adhesions. In addition, within the PC3 cell population there are some elongated cells, whereas the HeLa cell populations are more regular in shape with very few elongated cells under normal conditions (Figures 1 and 6). PC3 cells frequently became very elongated with long protrusions in response to PMM knockdown (Figure 1; Additional file 2, Figure S1), and *FAM40B, FMNL3 *and *FNBP3 *depletion induced elongation of PC3 cells (Figure 1) but not in HeLa cells (Figure 6). *FAM40B*-depleted HeLa cells had very bright regions of F-actin staining around the periphery associated with lamellipodia and filopodia, but very few thick cortical F-actin bundles. In addition, *FAM40B*-depleted HeLa cells appeared to detach from each other and had reduced cell-cell adhesion (Figure 7A). A reduction in cell-cell adhesion was also apparent in the scratch wound images from *FAM40B*-depleted PC3 cells (Figure 3; Additional file 11, movie 7). *FMNL3 *depletion did not have a strong effect on HeLa cell morphology, which might reflect differences in the relative levels of FMNL3 in HeLa and PC3 cells compared to *FMNL1 *and *FMNL2*. Finally, knockdown of *FNBP3 *in HeLa cells appeared to inhibit cytokinesis, since many cells were binucleate (Figure 7C). In addition, cells had multiple filopodia/microspikes, both linking between cells and at cell edges away from cell-cell interactions (Figure 7B). Like FAM40B knockdown, this could indicate a reduction in cell-cell adhesion.

### Over-expression of FAM40A and FAM40B induces actin reorganization

**Figure 8:**
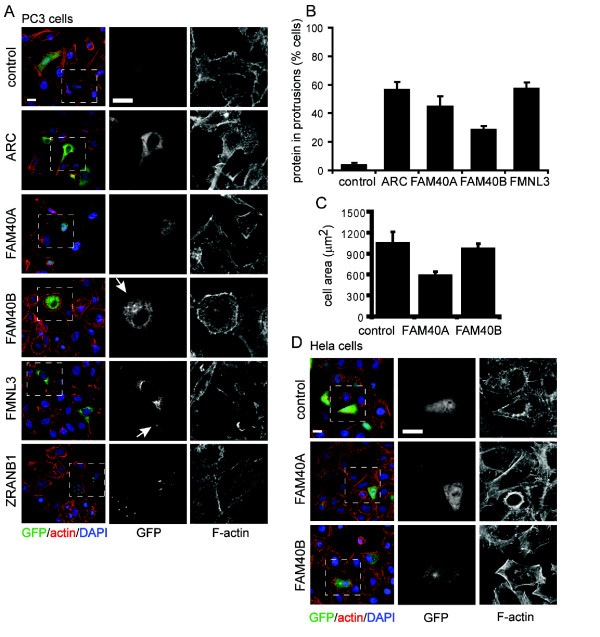
**PMM overexpression alters actin organization**. PC3 cells **(A) **or HeLa cells **(D) **plated on Matrigel were transfected with constructs encoding the indicated N-terminal GFP-tagged PMM proteins (ARC, FAM40A, FAM40B, FMNL3 and ZRANB1) or GFP alone (control). After 18 h, cells were fixed and stained for F-actin, α-tubulin and DNA (DAPI). Scale bars, 10 μm. Boxed areas in merged images are enlarged to show detail of F-actin and PMM localization. Arrowheads (A) indicate FAM40B and FMNL3 localization to protrusions. **(B)** Quantification of PMM protein localization in protrusions in transfected PC3 cells. Control is GFP alone. Values are means ± S.D. of 26 to 66 cells per condition in each of three independent experiments. **(C)** Cell area (μm^2^) of transfected PC3 cells. Values are means ± s.e.m. of 50 to 100 cells in three independent experiments. ***P ≤ 0.001, control vs. FAM40A (unpaired Student's *t*-test).

To investigate how overexpression of selected PMMs affected cell morphology and cytoskeletal organisation and to determine their localization, cDNAs for *ARC*, *FAM40A, FAM40B, FMNL3 *and *ZRANB1 *were cloned downstream of GFP and transfected into PC3 cells (Figure 8A). GFP-ARC, FAM40A, FAM40B and FMNL3 proteins showed cytoplasmic localization with some enrichment on the plasma membrane compared to GFP alone (Figure 8B). In particular, FMNL3 and FAM40B were localized in lamellipodia. In contrast, ZRANB1 (also known as TRABID) showed punctate localization in the cytoplasm, similar to a previous report [9]. Expression of FAM40A reduced PC3 cell area compared with control cells (Figure 8A, C), similar to knockdown of FAM40A, suggesting that FAM40A levels are critical for regulating cell spreading. In HeLa cells, expression of FAM40B increased cortical F-actin bundles (Figure 8D), consistent with the decrease of thick cortical F-actin bundles observed in FAM40B-depleted HeLa cells (Figure 6). The other PMMs had no consistent effect on cell shape or actin filament distribution.

## Discussion

Here we identify multiple novel regulators of cell morphology, cytoskeletal organisation and migration, many of which are implicated in human diseases and signal transduction processes. Sixteen genes were chosen based on their identification in a genome-wide *Drosophila *cell morphology screen followed by bioinformatic analysis of their domain structure, interacting partners and potential functions in other species. Of the 26 human genes identified as homologues of these *Drosophila *genes, nearly all affected cell shape and/or migration in PC3 prostate carcinoma cells, implying that these genes have conserved functions between *Drosophila *and human cells. Based on morphological analysis, these genes could be divided into six distinct groups, and this grouping was reflected in their effects on migration. This could in the future allow prediction of migratory behaviour based on cytoskeletal and shape analysis.

The PMM genes have not previously been identified in RNAi-based migration screens; for example, they were not in the gene set analysed by Simpson *et al*. [5] because they are not kinases, phosphatases or previously known regulators of cell migration. They were not identified in another migration screen of 5,234 genes [6], but the complete gene list is not provided for this screen.

Of the seven PMM genes chosen for in-depth characterization, the closely related *FAM40A *and *FAM40B *genes had clearly distinct phenotypes: *FAM40A*-depleted cells had an increase in F-actin around the periphery and a circular shape, whereas *FAM40B*-depleted cells were highly elongated. However, knockdown of each of these genes only led to a mild decrease in migration in scratch wounds. Indeed, other genes that induced cell elongation in PC3 cells did not affect migration, indicating that under the conditions we have analysed elongation is not predictive of a cell migration defect. Very little is known about *FAM40 *genes but interestingly they are conserved from fungi and amoebae to humans, although we have been unable to find a homologue in plants. The yeast homologue is FAR11 (pheromone arrest 11), which in *S. cerevisiae *interacts with other FAR proteins and causes a Far3-like pheromone arrest phenotype when disrupted [19]. Protein-protein interaction databases report that FAR11 interacts with Rom2, a GEF for the Rho GTPases Rho1 and Rho2 (BioGRID; [20]), as well as Rho4 and Tpd3 [19]. In human cells, FAM40A and FAM40B have recently been isolated as part of a **str**iatin-**i**nteracting **p**hosphatase **a**nd **k**inase (STRIPAK) complex, that also contains PP2A phosphatases, striatins, MOB1/3 and members of the Ste20 kinase family [21]. Interestingly, *S. cerevisiae *Far8 shares sequence similarity with the striatins, and Tpd3, a PP2A-A orthologue, has been found as an interacting partner with Far11 in yeast two-hybrid assay [19]. Altogether these data suggest that FAM40A and FAM40B could be part of a protein complex conserved from yeast to humans. Functions of the STRIPAK complex are still to be determined but it is possible it plays a role in intracellular trafficking, and/or in cytoskeletal changes, since yeast MOB1 is involved in cytoskeletal reorganization during exit from mitosis and cytokinesis [22].

FMNL1, 2 and 3 are part of the Diaphanous-related formin family of proteins, several of which have been shown to be regulated by Rho GTPases [23,24]. Depletion of each of the three FMNL proteins induced an increase in peripheral F-actin rich lamellipodial protrusions in PC3 cells, but only FMNL3 knockdown significantly inhibited migration in scratch wounds. FMNL1/FRLα has been shown to stimulate actin polymerization, bind to Rac1, and regulate macrophage motility [25,26]. FMNL2 and FMNL3 have recently been reported to bind to RhoC and regulate migration and invasion [27,28]. Our observation that FMNL3 severely impairs migration and localizes in part to lamellipodia is in accordance with these observations.

Formin family proteins have multiple binding partners, known as formin-binding proteins (FBPs or FNBPs). It is interesting that we identified that depletion of a FBP, FNBP3, strongly inhibits migration, similar to FMNL3 knockdown. Its close relative HYPC did not have a marked effect on migration or morphology of PC3 cells, which might reflect low-level expression compared to FNBP3. FNBP3 (also known as FBP11/PRPF40A) was identified in a screen for formin-1-binding proteins, which identified multiple proteins with WW domains [29,30]. However, of these WW domain-containing FBPs, only FBP17 has been characterized in detail [23]. FNBP3 and HYPC are orthologues of the *S. cerevisiae *splicing factor PRP40, but whether they have a role in splicing in mammals is not known. FNBP3 has been reported to interact with N-WASP and inhibit its translocation to the cytoplasm [31] and both FNBP3 and HYPC interact with the N-terminus of Huntingtin (HTT) [32], but little is known of the functional relevance of these interactions. Our results, showing that *FNBP3 *depletion strongly inhibits cell migration in PC3 cells and cytokinesis in HeLa cells, imply that FNBP3 is likely to act through a formin family protein, which is known to affect cell protrusion and cytokinesis [24,33]. It would be interesting to know whether FNBP3 and FMNL3 act together to regulate cell migration, although only FNBP3 has an effect on cytokinesis so would be expected to act through a different formin for this process.

LIMD1 and WTIP are members of the LIM domain-containing the Ajuba/Zyxin family. Several members of the family, such as Zyxin, are involved in the regulation of cell adhesion and also translocate to the nucleus where they regulate gene expression [34]. LIMD1 and WTIP also appear to be multifunctional proteins. LIMD1 is implicated in osteoclast development through effects on transcription factor activity [35], but has also been reported to localise to focal adhesions [36]. WTIP interacts with and affects the transcriptional activity of Wilm's tumour protein, yet also localizes to cell-cell junctions and podocytes in kidney cells [37], and interacts with the receptor tyrosine kinase ROR2 [38]. LIMD1 and WTIP depletion induce distinct changes in actin organization: *LIMD1 *knockdown leads to an increase in peripheral F-actin bundles and stress fibres (in HeLa cells), whereas *WTIP *knockdown induces the formation of small F-actin-rich protrusions. These effects could be mediated by nuclear or cytoplasmic functions of LIMD1 and WTIP.

Unlike the other PMM genes we analysed in detail, *ARC *(activity-regulated cytoskeleton-associated protein) and *ZRANB1 *are each unique genes without closely related isoforms in mammals. Depletion of each of them strongly inhibits migration of PC3 cells and, like *LIMD1 *knockdown, increased stress fibres. *ARC *was originally identified as a gene rapidly induced by neuronal activity [10], and mice lacking *ARC *have deficits in long-term memory [39,40]. ARC has also been implicated in endocytosis through interactions with endophilin and dynamin [41]. Our data indicate for the first time that it plays an important role in cancer cell migration and actin cytoskeletal organization, which could be linked to endocytosis, although it does not specifically localize to vesicular structures.

ZRANB1 (also known as TRABID) has three zinc-finger domains and a ubiquitin thioesterase domain, and has been reported to de-ubiquitinate APC and, thereby, regulate Wnt signalling [9]. It also binds to TRAF6, which in turn associates with TNF-receptor family members [42]. It is possible that it regulates cell migration and stress fibres through effects on APC ubiquitination, which is known to affect microtubules and the actin cytoskeleton [43], although it could also deubiquitinate other proteins involved in cell migration.

## Conclusions

Our results indicate that multiple proteins affecting cell shape and cytoskeletal organization are conserved from *Drosophila *to human cells, and that they can be divided into groups that reflect their morphology and effects on migration. Where one *Drosophila *gene has two or more closely related paralogues in humans, the two do not act redundantly. Instead, each of the isoforms has a distinct effect on cell shape, implying divergence of functions. Knockdown of several proteins increases stress fibres and inhibits cell migration, consistent with other data indicating an inverse correlation between stress fibre levels and migration speed [44,45]. Other proteins that regulate migration speed act through changes in the shape and size of cell protrusions such as lamellipodia, which is known to alter migration efficiency [1]. These data support our approach as an effective strategy by which to identify new regulators of cytoskeletal organisation and cell migration. Combining the morphological information with bioinformatic analysis has allowed us to identify new links between cell shape and genes previously implicated in a variety of human diseases, including Huntington's disease, Wilm's tumour, mental retardation, and Down syndrome.

## Methods

### Analysis of hits using genomic databases

A database containing images from a *Drosophila melanogaster *S2R+ cell genome-wide RNAi morphology screen, FLIGHT [46], was used to screen images manually to identify genes that affected actin filament or microtubule distribution and/or cell shape. Genes without a well-described function in *Drosophila *were chosen for further analysis. A bioinformatic approach was used to gather information on their potential functions, using similarity of protein domains and data available from orthologues and/or paralogues in other species. This included information from protein-protein interaction screens obtained from multiple databases including Fly General Repository for Interaction Datasets (Fly GRID), Human Protein Reference Database (HPRD), PubMed Gene, and Genecards. This led to the selection of 16 conserved *Drosophila *genes, for which 26 human genes were identified (26 PMMs) based on homology.

### Cell culture and siRNA transfection

The prostate cancer-derived cell line PC3 (a gift from Prof J Masters, University College London) was grown in RPMI containing 25 mM Hepes and 2 mM glutamine supplemented with 10% fetal bovine serum (FBS), 100 U/ml penicillin and 100 μg/ml streptomycin. HeLa (Kyoto strain) and NIH3T3 cells were cultured in Dulbecco's Modified Eagle Medium (Invitrogen, Paisley, UK[) supplemented with 10% FBS, 50 U/ml penicillin and 50 μg/ml streptomycin.

Four individual siRNAs (Dharmacon siGENOME duplexes, ThermoFisher Scientific, Loughborough, UK) were purchased for each gene, and then pooled (Additional file 14, Table S3). The control siRNA was a SMARTpool of four non-targeting siRNAs (Dharmacon D-001206-13). For the PMM screens, PC3 cells were reverse-transfected in black, thin-bottomed 384-well tissue-culture plates suitable for confocal imaging (2,500 cells per well) (Greiner Bio-One, Stonehouse, UK) with 25 to 100 nM of siRNA pools using Lipofectamine-2000 (Invitrogen, Paisley, UK) according to the manufacturer's instructions. For high-resolution confocal imaging, PC3 cells were transfected on Matrigel-coated (100 μg/ml) eight-well chambers (Becton Dickinson, Oxford, UK) or glass coverslips. HeLa cells (2,000 cells per well) were reverse-transfected in black 384-well tissue-culture plates at a final concentration of 25 nM of siRNA pools. PC3 and HeLa cells were analysed 72 h after transfection.

### cDNA cloning and plasmid transfection

The open reading frames for *ARC, FAM40A, FAM40B, FMNL3 *and *ZRANB *were PCR amplified either from cDNA clones or primary cDNA and subsequently cloned by Gateway™ (Invitrogen, Paisley, UK) BP reaction into the Gateway™ vector pDONR221 as described [47]. The resulting entry clones were completely sequence verified and used for the generation of GFP-tagged PMMs. In brief, 150 ng of each entry clone was recombined with 150 ng of destination vector, pcDNA-DEST53 or pcDNA-DEST47, to generate N-terminal or C-terminal GFP-tagged constructs, respectively. Positive clones growing on ampicillin were selected and were verified by enzymatic restriction using BsrGI. N-terminal GFP-tagged PMMs were generally expressed at a higher level and were used for further experiments. For transfection, cells were grown on Matrigel-coated (100 μg/ml) eight-well chambers. They were transfected with plasmid DNA (0.6 μg) using Lipofectamine-2000 according to the manufacturer's instructions, and fixed and stained after 18 h.

### Immunofluorescence, confocal microscopy and image analysis

For fluorescence staining, cells were fixed with 4% paraformaldehyde, permeabilized with 0.1% or 0.2% Triton X-100 in PBS and blocked with 1% BSA or 5% FBS (PC3 cells) or 5% BSA (HeLa cells) in PBS. Cells were stained with DAPI (1 μg/ml, Sigma-Aldrich, Gillingham, UK) for nuclear staining, Alexa Fluor-conjugated phalloidin (1 μM, wavelengths 480 nm, 543 nm, 546 nm or 633 nm, Molecular Probes/Invitrogen, Paisley, UK) or TRITC-conjugated phalloidin (0.125 μg/ml, Sigma) for F-actin visualization, and 1:400 FITC-labelled α-tubulin antibody (DM1A clone; Sigma) for microtubules. Images were acquired by automated microscopy on a Nikon TE2000 microscope (Kingston, UK) with a 20× objective. Confocal images were acquired with a Zeiss LSM 510 (Welwyn Garden City, UK) or a Leica SP5 (Milton Keynes, UK) inverted confocal microscope.

Morphology analysis was carried out using Image J software http://rsbweb.nih.gov/ij/index.html. Circularity was determined with the Circularity Plugin. The area of PC3 cells transfected with constructs encoding GFP-fusion proteins was determined from F-actin-stained fluorescence images. For stress fibre quantification, images of NIH3T3 cells expressing GFP-fusion proteins and stained for F-actin were magnified electronically, and cells were classified visually based on their content of stress fibres.

### Time-lapse microscopy and cell tracking

For time-lapse microscopy, a fully motorized, multi-field Nikon TE2000 microscope was used. PC3 cells were reverse transfected in 96-well plates (17,000 cells/well) with 50 nM siRNA pools using Lipofectamine-2000. For scratch wound migration, scratch wounds were created in the centre of the cell monolayer 72 h after transfection, using a micropipette tip. After washing twice with culture medium, phase contrast cell images were acquired every 30 minutes for 16 h (10× objective). Wound healing quantification was carried out using custom-written Metamorph journals. The area covered by cells on the two sides of the wound was detected by locating the plasma membrane (using a gradient filter) and filling the space surrounded by plasma membrane (using a morphological dilation transformation). The surface areas covered by the cells in the successive frames were exported into Excel to measure wound healing speed. The speed was estimated by applying a linear fitting to the area series using the "SLOPE" function (Excel, Microsoft, Reading, UK). The resulting wound healing speed is expressed in μm/min.

For random migration, cells were seeded on Matrigel-coated 24-well plates 48 h after transfection. After 24 h images were acquired every 10 minutes for 14 h. Cells were tracked using ImageJ Manual tracking and Chemotaxis tool plugins.

## Abbreviations

APC: adenomatous polyposis coli protein; BSA: bovine serum albumin; DAPI: 4',6-diamidino-2-phenylindole; FBP: formin-binding protein; FBS: fetal bovine serum; GFP: green fluorescent protein; GEF: guanine nucleotide exchange factor; PBS: phosphate-buffered saline; PMMs: putative motility modifiers; siRNA: small interfering RNA; TNF: tumour necrosis factor.

## Competing interests

The authors declare that they have no competing interests.

## Authors' contributions

SWB designed experiments, performed most of the experiments and produced most of the figures. MTHA and VT cloned PMMs into GFP vectors and analysed their effects. JLR performed experiments with HeLa cells. VR wrote software for analysing scratch-wound migration and helped with statistical analysis. NS assisted with cell tracking, while SB cloned PMMs into entry vectors, and SW supervised SB and generated entry vector cDNA libraries. BB supervised analysis of *Drosophila *screen images and the work of JLR, and contributed to experimental design. AR supervised the project, designed experiments and wrote the manuscript with contributions from SWB, MTHA, JLR, SW and BB. All authors read and approved the final manuscript.

## Supplementary Material

Additional file 1 **Table S1**. Selection of PMMs. Data on gene names, alternative names, domains and interaction partners are taken from NCBI Gene information and/or GeneCards http://www.genecards.org; x, no information available. Click here for file

Additional file 2 **Figure S1**. Effects of PMM depletion on cell morphology and cytoskeletal organization. (**A**) PC3 cells were transfected with the indicated siRNA pools for each PMM in 384-well plates. (**B**) PC3 cells plated on Matrigel were transfected with the indicated siRNA pools. Cells were fixed after 72 h, then stained for F-actin (red), α-tubulin (green) and nuclei (DAPI, blue). Images in A were acquired on an automated Nikon microscope; images in B were acquired by confocal microscopy. Scale bar, 100 μm (A); 10 μm (B). Click here for file

Additional file 3 **Figure S2**. Examples of actin and shape phenotypes in PMM-depleted cells. Description: Images show examples of cytoskeletal and shape phenotypes used to classify PMMs into groups in Table 2. PC3 cells were transfected with siRNA pools targeting ARC (stress fibres), FMNL1 (cortical actin), PDZK8 (actin patches), ZMYM4 (very elongated), FAM40B (elongated), or FMNL2 (processes). Images shown are taken from the images in Figure S1, and show the F-actin or microtubule channels separately as indicated. Arrows indicate examples of stress fibres, cortical actin, actin patches or processes. Click here for file

Additional file 4 **Table S2**. Phenotypes induced by individual siRNAs. Description: Each of four different siRNA oligos targeting the indicated genes (listed in Table S2) was transfected into PC3 cells or HeLa cells. The number of oligos that gave the same morphological phenotype as the pool of four oligos (Figure 1 and Table S2 for PC3, Figure 6 for HeLa cells) is shown. NP, no phenotype; x, not tested (weak phenotype with pool). Click here for file

Additional file 5 **Movie 1**. Control siRNA. PC3 cells were transfected with siRNA pools for each PMM in 96-well plates. After 72 h, cells were scratch wounded and then monitored by time-lapse microscopy. Phase contrast cell images were acquired every 30 minutes for 16 hours. Click here for file

Additional file 6 **Movie 2**. ZRANB1 siRNA. PC3 cells were transfected with siRNA pools for each PMM in 96-well plates. After 72 h, cells were scratch wounded and then monitored by time-lapse microscopy. Phase contrast cell images were acquired every 30 minutes for 16 hours. Click here for file

Additional file 7 **Movie 3**. *ARC *siRNA. PC3 cells were transfected with siRNA pools for each PMM in 96-well plates. After 72 h, cells were scratch wounded and then monitored by time-lapse microscopy. Phase contrast cell images were acquired every 30 minutes for 16 hours. Click here for file

Additional file 8 **Movie 4**. *FMNL3 *siRNA. PC3 cells were transfected with siRNA pools for each PMM in 96-well plates. After 72 h, cells were scratch wounded and then monitored by time-lapse microscopy. Phase contrast cell images were acquired every 30 minutes for 16 hours. Click here for file

Additional file 9 **Movie 5**. *FNBP3 *siRNA. PC3 cells were transfected with siRNA pools for each PMM in 96-well plates. After 72 h, cells were scratch wounded and then monitored by time-lapse microscopy. Phase contrast cell images were acquired every 30 minutes for 16 hours. Click here for file

Additional file 10 **Movie 6**. *LIMD1 *siRNA. PC3 cells were transfected with siRNA pools for each PMM in 96-well plates. After 72 h, cells were scratch wounded and then monitored by time-lapse microscopy. Phase contrast cell images were acquired every 30 minutes for 16 hours. Click here for file

Additional file 11 **Movie 7**. *FAM40B *siRNA. PC3 cells were transfected with siRNA pools for each PMM in 96-well plates. After 72 h, cells were scratch wounded and then monitored by time-lapse microscopy. Phase contrast cell images were acquired every 30 minutes for 16 hours. Click here for file

Additional file 12 **Movie 8**. *FAM40A *siRNA. PC3 cells were transfected with siRNA pools for each PMM in 96-well plates. After 72 h, cells were scratch wounded and then monitored by time-lapse microscopy. Phase contrast cell images were acquired every 30 minutes for 16 hours. Click here for file

Additional file 13 **Figure S3**. Migration speeds of PMM-depleted PC3 cells. PC3 cells transfected with siRNAs for each of the indicated PMMs were imaged by time-lapse analysis for 14 h. Cells were tracked (Figure 3) and speeds determined. A total of 70 to 99 cells were tracked from three movies for each PMM. Values are means +/- s.e.m.; *** *P *≤ 0.001, compared to control siRNA-transfected cells (unpaired Student's *t*-test). Click here for file

Additional file 14 **Table S3**. siRNAs used for experiments. The names of the 26 human PMM genes and actin regulatory genes, and 4 siRNA sequences (sense strand) that were used as a pool for knockdown of each gene are shown. Only one siRNA was used for RhoA. Click here for file
