# Supplementary material for: Identification and characterization of a set of conserved and new regulators of cytoskeletal organization, cell morphology and migration
Source: BMC Biol. 2011 Aug 11;9:54. doi: 10.1186/1741-7007-9-54 (PMC3201212; doi:10.1186/1741-7007-9-54)
Supplement: Additional file 1 — Table S1. Selection of PMMs. Data on gene names, alternative names, domains and interaction partners are taken from NCBI Gene information and/or GeneCards http://www.genecards.org; x, no information available. [file 1741-7007-9-54-S1.DOC]

| **Drosophila gene** | **Drosophila RNAi screens** | **Human gene symbol** | **Entrez Gene ID** | **Functional data** | **Domains** | **Protein interactions** |
| --- | --- | --- | --- | --- | --- | --- |
| CG12505 | x | ARC | 23237 | Co-precipitates with F-actin, enriched in neuronal dendrites; linked with endocytosis, F-actin formation underlying synaptic plasticity, memory (Bramham et al., 2008). | x | Dynamin, SH3GL2 (Chowdhury et al., 2006), spectrin, nuclear PML (Bloomer et al., 2007), keratin 15 (Rual et al., 2005) |
| CG31132 | JAK/STAT signalling | BRWD1 | 54014 | Human gene located within Down syndrome region-2, chromosome 21; putative transcription factor associated with SWI/SNF chromatin remodeling (Huang et al., 2003); required for gametogenesis (Philipps et al., 2008). | WD-40 repeat, bromo  domain | SMARCA4/BRG1 (Huang et al., 2003) |
|  |  | BRWD3 | 254065 | Mutated in X-linked mental retardation (Field et al., 2007). | WD-40 repeat, bromo  domain | UBXD7 (Alexandru et al., 2008) |
|  |  | PHIP | 55023 | Regulates insulin and IGF-1 signaling (Farhang-Fallah et al., 2000; Goodwin and Vale, 2010). | WD-40 repeat, Peptidase S14, ClpP | IRS1 (Farhang-Fallah et al., 2000), STMN4, TUBB5 (Stelzl et al., 2005) |
| CG10671 | x | C20orf142 | 128486 | Member of protein family involved in fat storage; located in endoplasmic reticulum membrane (Kadereit et al., 2008). | multiple potential trans  membrane domains | x |
| CG14995 | growth and viability; mycobacterial infection; Wnt-Wingless signalling | C21orf2 | 755 | Linked with Down syndrome (Shim et al., 2003). | leucine-rich repeats; U2A' phosphor  protein32A_C | PFKL; MX1; AIRE ([http://www.genecards](http://www.genecards/).  org/) In Drosophila: Myo10A, Importin-a2 (http://flight.licr.org/) |
| CG33130 | cell size and cell cycle progression | CAMSAP1 | 157922 | Localizes to microtubules (Baines et al., 2009); Drosophila homologue ssp4/patronin stabilizes microtubule minus ends (Goodwin and Vale, 2010), | CKK, CH | SKIL, SMAD1, SMAD9, CAMSAP1L1 (Colland et al., 2004) |
| CG11339 | x | EPB41L4A | 64097 | Induced by ß-catenin/Tcf (Ishiguro et al., 2000). | Band 4.1; ERM; FERM-type; PH_type. | ND |
| CG11526 | Compartmentali-zation of intracellular pathogen | FAM40A | 85369 | Components of STRIPAK complex (Goudreault et al., 2009); S. cerevisiae homologue FAR11 involved in pheromone response pathway ((Kemp and Sprague, 2003) and interacts with Rho4; C. elegans homologue F10E7.8 linked with dynein (O'Rourke et al., 2007); N. crassa homologue ham-2 required for hyphal fusion (Xiang et al., 2002). | N1221-like | CCM3, CTTNBP2, FGFR1OP2, MOBKL3, MST4, PPP2R1A, PP2CB, SIKE1, STK24, STK25, STRN3, STRN4, TRAFIP3 (Goudreault et al., 2009) |
|  |  | FAM40B | 57464 |  |  |  |
| CG32138 | x | FMNL1 | 752 | Formin-related proteins regulate actin polymerization (Schonichen and Geyer, 2010); FMNL1 regulates macrophage podosomes and motility (Mersich et al.; Yayoshi-Yamamoto et al., 2000) | DAD, FH2, GBD/FH3,  ARM-like | Rac1, Profilin (Yayoshi-Yamamoto et al., 2000), FNBP3, Ku antigen (Stelzl et al., 2005), Akt (Favaro et al., 2006), SrGAP2 (Mason et al., 2010) |
|  |  | FMNL2 | 114793 |  |  | Cdc42 (Bandyopadhyay et al., 2010), FNBP3 (Colland et al., 2004) |
|  |  | FMNL3 | 91010 |  |  | FNBP3 (Bedford et al., 1997) |
| CG3542 | innate immune response | FNBP3 | 55660 | May be involved in pre-mRNA splicing (Lin et al., 2004); localizes to nuclear speckles; linked with N-WASP (Mizutani et al., 2004); linked to Huntington’s disease (Passani et al., 2000) | 2 WW, 2 FF, PRP40 | Many proteins including Huntingtin (Faber et al., 1998), formins (Bedford et al., 1997), MeCP2 (Buschdorf and Stratling, 2004), AKAP8L (Sayer et al., 2005), ARHGEF2 (Colland et al., 2004) |
|  |  | HYPC | 25766 | May be involved in pre-mRNA splicing (Faber et al., 1998); linked to Huntington’s disease (Passani et al., 2000) |  | Huntingtin (Faber et al., 1998), MeCP2 (Buschdorf and Stratling, 2004) |
| CG11505 | cell size and cell cycle progression,  JAK/STAT signalling | LARP4 | 113251 | Binds poly(A), promotes mRNA stability (Yang et al., 2010); S. cerevisiae homologue SRO9 associates with translating ribosomes (Sobel and Wolin, 1999); SRO9 affects actin organization (Kagami et al., 1997) | HTH La-type RNA-bd, RRM_RNP | PABP, RACK1, RNA (Yang et al., 2010) |
| CG11063 | x | LIMD1 | 8994 | Member of zyxin family; localizes to focal adhesions (Huggins and Andrulis, 2008; Petit et al., 2005), adherens junctions, and nucleus where it acts as a retinoblastoma protein corepressor (Sharp et al., 2004); tumor suppressor in cancer (Sharp et al., 2008; Spendlove et al., 2008); LIMD1, WTIP and relative Ajuba regulate miRNA-mediated gene silencing (James et al., 2010); | LIM zinc-binding | Retinoblastoma protein (Sharp et al., 2004), HIPK3 (Colland et al., 2004) |
|  |  | WTIP | 126374 | Member of zyxin family; podocyte adherens junction protein, and regulator of WT1-dependent transcription (Srichai et al., 2004); stress signaling pathways induce WTIP nuclear translocation (Kim et al., 2010). |  | WT1 (Srichai et al., 2004), ROR2 (van Wijk et al., 2009) |
| CG10362 | x | PDZK8 | 118987 | Promotes HIV infection (Henning et al., 2010) | Prot_Kinase_C-like_PE/DAG-bd, PDZ | Gag of HIV-1 (Henning et al., 2010) |
| CG31012 | x | SH3D19 | 152503 | May regulate A disintegrin and metalloproteases (ADAMs)-mediated EGFR-ligand shedding (Tanaka et al., 2004) and Ras-induced cell transformation and ELK1 activation (Yam et al., 2004); RUNX1-SH3D19 translocation in leukaemia (Nguyen et al., 2006) | 5 SH3, p67phox | ADAMs (Tanaka et al., 2004), SH3YL1(Shimomura et al., 2003), SOS2, SH3GL1 and 3 (Tanaka et al., 2004) |
|  |  | SH3KBP1 | 30011 | Signal transduction adapter protein; regulates receptor trafficking and degradation, cell adhesion and migration (reviewed in(Havrylov et al., 2010)) | 3 SH3, p67phox | Many proteins including CBL (Soubeyran et al., 2002), GRB2 (Borinstein et al., 2000), PAK2 (Kurakin et al., 2003) |
| CG5965/ woc | x | ZMYM3 | 9203 | Candidate gene for X-linked mental retardation (van der Maarel et al., 1996); putative component of BHC histone deacetylase complex (Hakimi et al., 2003) | TRASH; Znf_MYM 9 MYM-type zinc fingers | KDM1A/BHC110, HDAC2 (Hakimi et al., 2003) |
|  |  | ZMYM4 | 9202 | mRNA 3'-UTR contains cell death-inhibiting RNA motif that binds AUF1 and inhibits interferon-γ-induced apoptosis (Shchors et al., 2004; Shchors et al., 2002) | TRASH; Znf_MYM | SUMO2 (Ouyang et al., 2009), ZMYM2 (Gocke and Yu, 2008), KLHL13 (Sowa et al., 2009) |
|  |  | ZMYM6 | 9204 | x | TRASH; Znf_MYM | CDK9 ([http://www.genecards](http://www.genecards/).  org/) |
| CG12299 | Wnt-Wingless signalling | ZNF-  135 | 7694 | Member of Krüppel transcription factor family (Tommerup and Vissing, 1995) | KRAB-A box; zf_C2H2/integrase_DNA-bd | x |
| CG9448 | cardiogenic genes | ZRANB1 | 54764 | Deubiquitinase; may modulate NFκB signaling (Evans et al., 2001); regulates Wnt signaling pathway, deubiquitinates APC (Tran et al., 2008) | OTU; Zf_RanBP2 | multiple proteins including TRAF6 (Evans et al., 2001), APC (Tran et al., 2008), STRIPAK complex (Sowa et al., 2009) |

**Supplementary Table 1. Selection of PMMs.** Data on human gene symbols and human gene names (HGNC), alternative names (aliases), domains and interaction partners are taken from NCBI Gene information and/or GeneCards (www.genecards.org); x, no information available.

**Supplementary references**

**Alexandru, G., Graumann, J., Smith, G. T., Kolawa, N. J., Fang, R. and Deshaies, R. J.** (2008). UBXD7 binds multiple ubiquitin ligases and implicates p97 in HIF1alpha turnover. *Cell* **134**, 804-816.

**Baines, A. J., Bignone, P. A., King, M. D., Maggs, A. M., Bennett, P. M., Pinder, J. C. and Phillips, G. W.** (2009). The CKK domain (DUF1781) binds microtubules and defines the CAMSAP/ssp4 family of animal proteins. *Mol. Biol. Evol.* **26**, 2005-2014.

**Bandyopadhyay, S., Chiang, C. Y., Srivastava, J., Gersten, M., White, S., Bell, R., Kurschner, C., Martin, C. H., Smoot, M., Sahasrabudhe, S. et al.** (2010). A human MAP kinase interactome. *Nat. Methods* **7**, 801-805.

**Bedford, M. T., Chan, D. C. and Leder, P.** (1997). FBP WW domains and the Abl SH3 domain bind to a specific class of proline-rich ligands. *EMBO J.* **16**, 2376-2383.

**Bloomer, W. A., VanDongen, H. M. and VanDongen, A. M.** (2007). Activity-regulated cytoskeleton-associated protein Arc/Arg3.1 binds to spectrin and associates with nuclear promyelocytic leukemia (PML) bodies. *Brain Res.* **1153**, 20-33.

**Borinstein, S. C., Hyatt, M. A., Sykes, V. W., Straub, R. E., Lipkowitz, S., Boulter, J. and Bogler, O.** (2000). SETA is a multifunctional adapter protein with three SH3 domains that binds Grb2, Cbl, and the novel SB1 proteins. *Cell Signal.* **12**, 769-779.

**Bramham, C. R., Worley, P. F., Moore, M. J. and Guzowski, J. F.** (2008). The immediate early gene arc/arg3.1: regulation, mechanisms, and function. *J. Neurosci.* **28**, 11760-11767.

**Buschdorf, J. P. and Stratling, W. H.** (2004). A WW domain binding region in methyl-CpG-binding protein MeCP2: impact on Rett syndrome. *J. Mol. Med.* **82**, 135-143.

**Chowdhury, S., Shepherd, J. D., Okuno, H., Lyford, G., Petralia, R. S., Plath, N., Kuhl, D., Huganir, R. L. and Worley, P. F.** (2006). Arc/Arg3.1 interacts with the endocytic machinery to regulate AMPA receptor trafficking. *Neuron* **52**, 445-459.

**Colland, F., Jacq, X., Trouplin, V., Mougin, C., Groizeleau, C., Hamburger, A., Meil, A., Wojcik, J., Legrain, P. and Gauthier, J. M.** (2004). Functional proteomics mapping of a human signaling pathway. *Genome Res.* **14**, 1324-1332.

**Evans, P. C., Taylor, E. R., Coadwell, J., Heyninck, K., Beyaert, R. and Kilshaw, P. J.** (2001). Isolation and characterization of two novel A20-like proteins. *Biochem J.* **357**, 617-623.

**Faber, P. W., Barnes, G. T., Srinidhi, J., Chen, J., Gusella, J. F. and MacDonald, M. E.** (1998). Huntingtin interacts with a family of WW domain proteins. *Hum. Mol. Genet.* **7**, 1463-1474.

**Farhang-Fallah, J., Yin, X., Trentin, G., Cheng, A. M. and Rozakis-Adcock, M.** (2000). Cloning and characterization of PHIP, a novel insulin receptor substrate-1 pleckstrin homology domain interacting protein. *J. Biol. Chem.* **275**, 40492-40497.

**Favaro, P. M., Traina, F., Vassallo, J., Brousset, P., Delsol, G., Costa, F. F. and Saad, S. T.** (2006). High expression of FMNL1 protein in T non-Hodgkin's lymphomas. *Leuk. Res.* **30**, 735-738.

**Field, M., Tarpey, P. S., Smith, R., Edkins, S., O'Meara, S., Stevens, C., Tofts, C., Teague, J., Butler, A., Dicks, E. et al.** (2007). Mutations in the BRWD3 gene cause X-linked mental retardation associated with macrocephaly. *Am. J. Hum. Genet.* **81**, 367-374.

**Gocke, C. B. and Yu, H.** (2008). ZNF198 stabilizes the LSD1-CoREST-HDAC1 complex on chromatin through its MYM-type zinc fingers. *PLoS One* **3**, e3255.

**Goodwin, S. S. and Vale, R. D.** (2010). Patronin regulates the microtubule network by protecting microtubule minus ends. *Cell* **143**, 263-274.

**Goudreault, M., D'Ambrosio, L. M., Kean, M. J., Mullin, M. J., Larsen, B. G., Sanchez, A., Chaudhry, S., Chen, G. I., Sicheri, F., Nesvizhskii, A. I. et al.** (2009). A PP2A phosphatase high density interaction network identifies a novel striatin-interacting phosphatase and kinase complex linked to the cerebral cavernous malformation 3 (CCM3) protein. *Mol. Cell. Proteomics* **8**, 157-171.

**Hakimi, M. A., Dong, Y., Lane, W. S., Speicher, D. W. and Shiekhattar, R.** (2003). A candidate X-linked mental retardation gene is a component of a new family of histone deacetylase-containing complexes. *J. Biol. Chem.* **278**, 7234-7239.

**Havrylov, S., Redowicz, M. J. and Buchman, V. L.** (2010). Emerging roles of Ruk/CIN85 in vesicle-mediated transport, adhesion, migration and malignancy. *Traffic* **11**, 721-731.

**Henning, M. S., Morham, S. G., Goff, S. P. and Naghavi, M. H.** (2010). PDZD8 is a novel Gag-interacting factor that promotes retroviral infection. *J. Virol.* **84**, 8990-8995.

**Huang, H., Rambaldi, I., Daniels, E. and Featherstone, M.** (2003). Expression of the Wdr9 gene and protein products during mouse development. *Dev. Dyn.* **227**, 608-614.

**Huggins, C. J. and Andrulis, I. L.** (2008). Cell cycle regulated phosphorylation of LIMD1 in cell lines and expression in human breast cancers. *Cancer Lett.* **267**, 55-66.

**Ishiguro, H., Furukawa, Y., Daigo, Y., Miyoshi, Y., Nagasawa, Y., Nishiwaki, T., Kawasoe, T., Fujita, M., Satoh, S., Miwa, N. et al.** (2000). Isolation and characterization of human NBL4, a gene involved in the ß-catenin/TCF signaling pathway. *Jpn. J. Cancer Res.* **91**, 597-603.

**James, V., Zhang, Y., Foxler, D. E., de Moor, C. H., Kong, Y. W., Webb, T. M., Self, T. J., Feng, Y., Lagos, D., Chu, C. Y. et al.** (2010). LIM-domain proteins, LIMD1, Ajuba, and WTIP are required for microRNA-mediated gene silencing. *Proc. Natl. Acad. Sci. U S A* **107**, 12499-12504.

**Kadereit, B., Kumar, P., Wang, W. J., Miranda, D., Snapp, E. L., Severina, N., Torregroza, I., Evans, T. and Silver, D. L.** (2008). Evolutionarily conserved gene family important for fat storage. *Proc. Natl. Acad. Sci. U S A* **105**, 94-99.

**Kagami, M., Toh-e, A. and Matsui, Y.** (1997). SRO9, a multicopy suppressor of the bud growth defect in the Saccharomyces cerevisiae rho3-deficient cells, shows strong genetic interactions with tropomyosin genes, suggesting its role in organization of the actin cytoskeleton. *Genetics* **147**, 1003-1016.

**Kemp, H. A. and Sprague, G. F., Jr.** (2003). Far3 and five interacting proteins prevent premature recovery from pheromone arrest in the budding yeast Saccharomyces cerevisiae. *Mol. Cell. Biol.* **23**, 1750-1763.

**Kim, J. H., Konieczkowski, M., Mukherjee, A., Schechtman, S., Khan, S., Schelling, J. R., Ross, M. D., Bruggeman, L. A. and Sedor, J. R.** (2010). Podocyte injury induces nuclear translocation of WTIP via microtubule-dependent transport. *J. Biol. Chem.* **285**, 9995-10004.

**Kurakin, A. V., Wu, S. and Bredesen, D. E.** (2003). Atypical recognition consensus of CIN85/SETA/Ruk SH3 domains revealed by target-assisted iterative screening. *J. Biol. Chem* .**278**, 34102-34109.

**Lin, K. T., Lu, R. M. and Tarn, W. Y.** (2004). The WW domain-containing proteins interact with the early spliceosome and participate in pre-mRNA splicing in vivo. *Mol. Cell. Biol.* **24**, 9176-9185.

**Mason, F. M., Heimsath, E. G., Higgs, H. N. and Soderling, S. H.** (2010). Bi-modal regulation of a formin by srGAP2. *J. Biol. Chem*., *in press*

**Mersich, A. T., Miller, M. R., Chkourko, H. and Blystone, S. D.** (2010). The formin FRL1 (FMNL1) is an essential component of macrophage podosomes. *Cytoskeleton (Hoboken)* **67**, 573-585.

**Mizutani, K., Suetsugu, S. and Takenawa, T.** (2004). FBP11 regulates nuclear localization of N-WASP and inhibits N-WASP-dependent microspike formation. *Biochem. Biophys. Res. Commun.* **313**, 468-474.

**Nguyen, T. T., Ma, L. N., Slovak, M. L., Bangs, C. D., Cherry, A. M. and Arber, D. A.** (2006). Identification of novel Runx1 (AML1) translocation partner genes SH3D19, YTHDf2, and ZNF687 in acute myeloid leukemia. *Genes Chromosomes Cancer* **45**, 918-932.

**O'Rourke, S. M., Dorfman, M. D., Carter, J. C. and Bowerman, B.** (2007). Dynein modifiers in C. elegans: light chains suppress conditional heavy chain mutants. *PLoS Genet.* **3**, e128.

**Ouyang, J., Shi, Y., Valin, A., Xuan, Y. and Gill, G.** (2009). Direct binding of CoREST1 to SUMO-2/3 contributes to gene-specific repression by the LSD1/CoREST1/HDAC complex. *Mol. Cell* **34**, 145-154.

**Passani, L. A., Bedford, M. T., Faber, P. W., McGinnis, K. M., Sharp, A. H., Gusella, J. F., Vonsattel, J. P. and MacDonald, M. E.** (2000). Huntingtin's WW domain partners in Huntington's disease post-mortem brain fulfill genetic criteria for direct involvement in Huntington's disease pathogenesis. *Hum. Mol. Genet.* **9**, 2175-2182.

**Petit, M. M., Crombez, K. R., Vervenne, H. B., Weyns, N. and Van de Ven, W. J.** (2005). The tumor suppressor Scrib selectively interacts with specific members of the zyxin family of proteins. *FEBS Lett.* **579**, 5061-5068.

**Philipps, D. L., Wigglesworth, K., Hartford, S. A., Sun, F., Pattabiraman, S., Schimenti, K., Handel, M., Eppig, J. J. and Schimenti, J. C.** (2008). The dual bromodomain and WD repeat-containing mouse protein BRWD1 is required for normal spermiogenesis and the oocyte-embryo transition. *Dev. Biol.* **317**, 72-82.

**Rual, J. F., Venkatesan, K., Hao, T., Hirozane-Kishikawa, T., Dricot, A., Li, N., Berriz, G. F., Gibbons, F. D., Dreze, M., Ayivi-Guedehoussou, N. et al.** (2005). Towards a proteome-scale map of the human protein-protein interaction network. *Nature* **437**, 1173-1178.

**Sayer, J. A., Manczak, M., Akileswaran, L., Reddy, P. H. and Coghlan, V. M.** (2005). Interaction of the nuclear matrix protein NAKAP with HypA and huntingtin: implications for nuclear toxicity in Huntington's disease pathogenesis. *Neuromolecular Med.* **7**, 297-310.

**Schonichen, A. and Geyer, M.** (2010). Fifteen formins for an actin filament: A molecular view on the regulation of human formins. *Biochim. Biophys. Acta* **1803**, 152-163.

**Sharp, T. V., Al-Attar, A., Foxler, D. E., Ding, L., de, A. V. T. Q., Zhang, Y., Nijmeh, H. S., Webb, T. M., Nicholson, A. G., Zhang, Q. et al.** (2008). The chromosome 3p21.3-encoded gene, LIMD1, is a critical tumor suppressor involved in human lung cancer development. *Proc. Natl. Acad. Sci. U S A* **105**, 19932-19937.

**Sharp, T. V., Munoz, F., Bourboulia, D., Presneau, N., Darai, E., Wang, H. W., Cannon, M., Butcher, D. N., Nicholson, A. G., Klein, G. et al.** (2004). LIM domains-containing protein 1 (LIMD1), a tumor suppressor encoded at chromosome 3p21.3, binds pRB and represses E2F-driven transcription. *Proc. Natl. Acad. Sci. U S A* **101**, 16531-16536.

**Shchors, K., Yehiely, F. and Deiss, L. P.** (2004). Cell Death Inhibiting RNA (CDIR) modulates IFN-gamma-stimulated sensitization to Fas/CD95/Apo-1 and TRAIL/Apo-2L-induced apoptosis. *Cell Cycle* **3**, 1606-1611.

**Shchors, K., Yehiely, F., Kular, R. K., Kotlo, K. U., Brewer, G. and Deiss, L. P.** (2002). Cell death inhibiting RNA (CDIR) derived from a 3'-untranslated region binds AUF1 and heat shock protein 27. *J. Biol. Chem.* **277**, 47061-47072.

**Shim, K. S., Bergelson, J. M., Furuse, M., Ovod, V., Krude, T. and Lubec, G.** (2003). Reduction of chromatin assembly factor 1 p60 and C21orf2 protein, encoded on chromosome 21, in Down syndrome brain. *J. Neural Transm. Suppl.*, 117-128.

**Shimomura, Y., Aoki, N., Ito, K. and Ito, M.** (2003). Gene expression of Sh3d19, a novel adaptor protein with five Src homology 3 domains, in anagen mouse hair follicles. *J. Dermatol Sci.* **31**, 43-51.

**Sobel, S. G. and Wolin, S. L.** (1999). Two yeast La motif-containing proteins are RNA-binding proteins that associate with polyribosomes. *Mol. Biol. Cell* **10**, 3849-3862.

**Soubeyran, P., Kowanetz, K., Szymkiewicz, I., Langdon, W. Y. and Dikic, I.** (2002). Cbl-CIN85-endophilin complex mediates ligand-induced downregulation of EGF receptors. *Nature* **416**, 183-187.

**Sowa, M. E., Bennett, E. J., Gygi, S. P. and Harper, J. W.** (2009). Defining the human deubiquitinating enzyme interaction landscape. *Cell* **138**, 389-403.

**Spendlove, I., Al-Attar, A., Watherstone, O., Webb, T. M., Ellis, I. O., Longmore, G. D. and Sharp, T. V.** (2008). Differential subcellular localisation of the tumour suppressor protein LIMD1 in breast cancer correlates with patient survival. *Int. J. Cancer* **123**, 2247-2253.

**Srichai, M. B., Konieczkowski, M., Padiyar, A., Konieczkowski, D. J., Mukherjee, A., Hayden, P. S., Kamat, S., El-Meanawy, M. A., Khan, S., Mundel, P. et al.** (2004). A WT1 co-regulator controls podocyte phenotype by shuttling between adhesion structures and nucleus. *J. Biol. Chem.* **279**, 14398-14408.

**Stelzl, U., Worm, U., Lalowski, M., Haenig, C., Brembeck, F. H., Goehler, H., Stroedicke, M., Zenkner, M., Schoenherr, A., Koeppen, S. et al.** (2005). A human protein-protein interaction network: a resource for annotating the proteome. *Cell* **122**, 957-968.

**Tanaka, M., Nanba, D., Mori, S., Shiba, F., Ishiguro, H., Yoshino, K., Matsuura, N. and Higashiyama, S.** (2004). ADAM binding protein Eve-1 is required for ectodomain shedding of epidermal growth factor receptor ligands. *J. Biol. Chem.* **279**, 41950-41959.

**Tommerup, N. and Vissing, H.** (1995). Isolation and fine mapping of 16 novel human zinc finger-encoding cDNAs identify putative candidate genes for developmental and malignant disorders. *Genomics* **27**, 259-264.

**Tran, H., Hamada, F., Schwarz-Romond, T. and Bienz, M.** (2008). Trabid, a new positive regulator of Wnt-induced transcription with preference for binding and cleaving K63-linked ubiquitin chains. *Genes Dev.* **22**, 528-542.

**van der Maarel, S. M., Scholten, I. H., Huber, I., Philippe, C., Suijkerbuijk, R. F., Gilgenkrantz, S., Kere, J., Cremers, F. P. and Ropers, H. H.** (1996). Cloning and characterization of DXS6673E, a candidate gene for X-linked mental retardation in Xq13.1. *Hum. Mol. Gene.t* **5**, 887-897.

**van Wijk, N. V., Witte, F., Feike, A. C., Schambony, A., Birchmeier, W., Mundlos, S. and Stricker, S.** (2009). The LIM domain protein Wtip interacts with the receptor tyrosine kinase Ror2 and inhibits canonical Wnt signalling. *Biochem. Biophys. Res. Commun.* **390**, 211-216.

**Xiang, Q., Rasmussen, C. and Glass, N. L.** (2002). The ham-2 locus, encoding a putative transmembrane protein, is required for hyphal fusion in Neurospora crassa. *Genetics* **160**, 169-180.

**Yam, J. W., Jin, D. Y., So, C. W. and Chan, L. C.** (2004). Identification and characterization of EBP, a novel EEN binding protein that inhibits Ras signaling and is recruited into the nucleus by the MLL-EEN fusion protein. *Blood* **103**, 1445-1453.

**Yang, R., Gaidamakov, S. A., Xie, J., Lee, J., Martino, L., Kozlov, G., Crawford, A. K., Russo, A. N., Conte, M. R., Gehring, K. et al.** (2011). LARP4 binds poly(A), interacts with poly(A)-binding protein MLLE domain via a variant PAM2w motif and can promote mRNA stability. *Mol. Cell. Biol*. **31,** 542-546.

**Yayoshi-Yamamoto, S., Taniuchi, I. and Watanabe, T.** (2000). FRL, a novel formin-related protein, binds to Rac and regulates cell motility and survival of macrophages. *Mol. Cell. Biol.* **20**, 6872-6881.
