# Supplementary figures and images for: Identification and characterization of a set of conserved and new regulators of cytoskeletal organization, cell morphology and migration
Source: BMC Biol. 2011 Aug 11;9:54. doi: 10.1186/1741-7007-9-54 (PMC3201212; doi:10.1186/1741-7007-9-54)

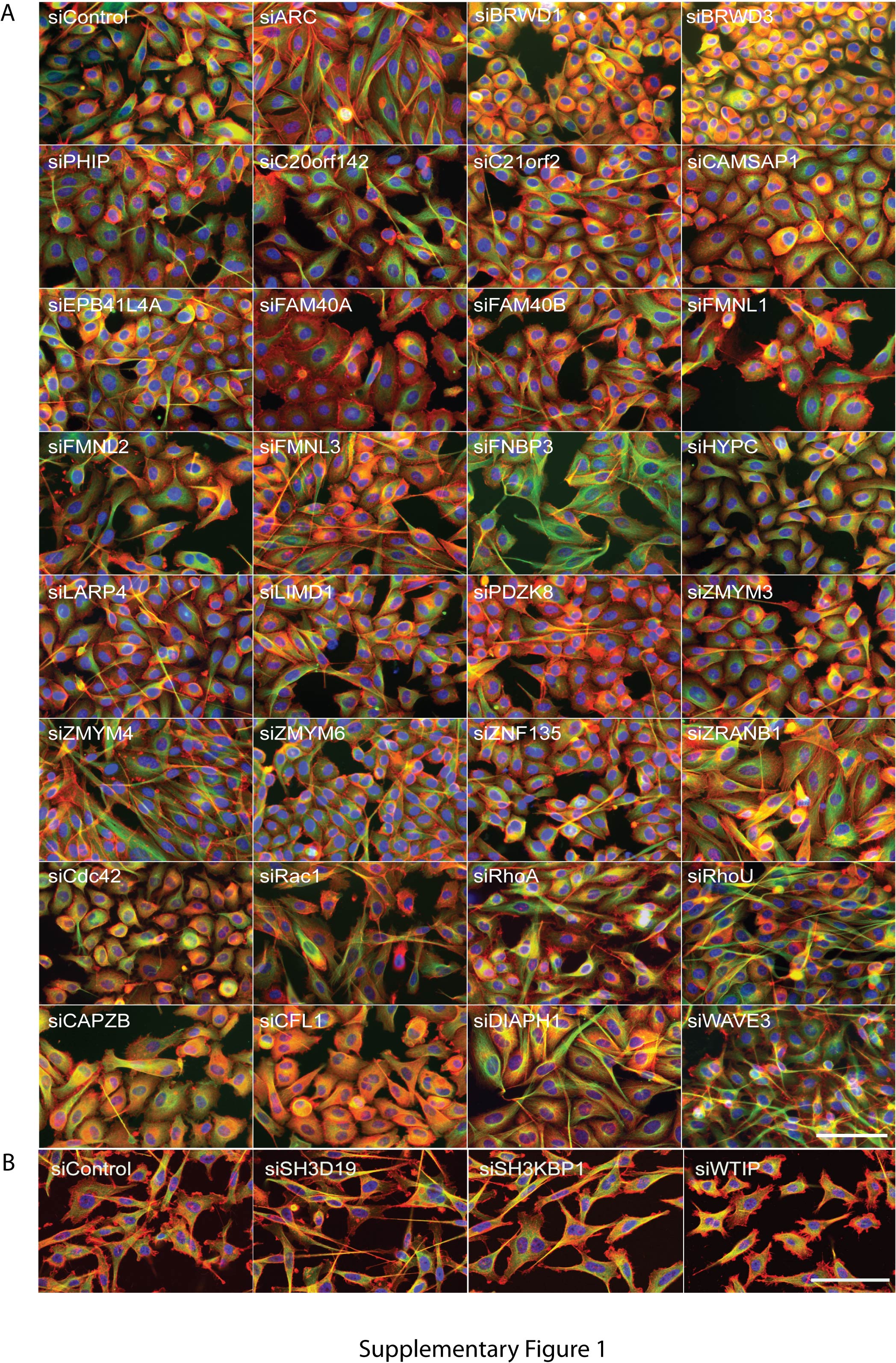

Supplement: Additional file 2 — Figure S1. Effects of PMM depletion on cell morphology and cytoskeletal organization. (A) PC3 cells were transfected with the indicated siRNA pools for each PMM in 384-well plates. (B) PC3 cells plated on Matrigel were transfected with the indicated siRNA pools. Cells were fixed after 72 h, then stained for F-actin (red), α-tubulin (green) and nuclei (DAPI, blue). Images in A were acquired on an automated Nikon microscope; images in B were acquired by confocal microscopy. Scale bar, 100 μm (A); 10 μm (B). [file 1741-7007-9-54-S2.JPEG]

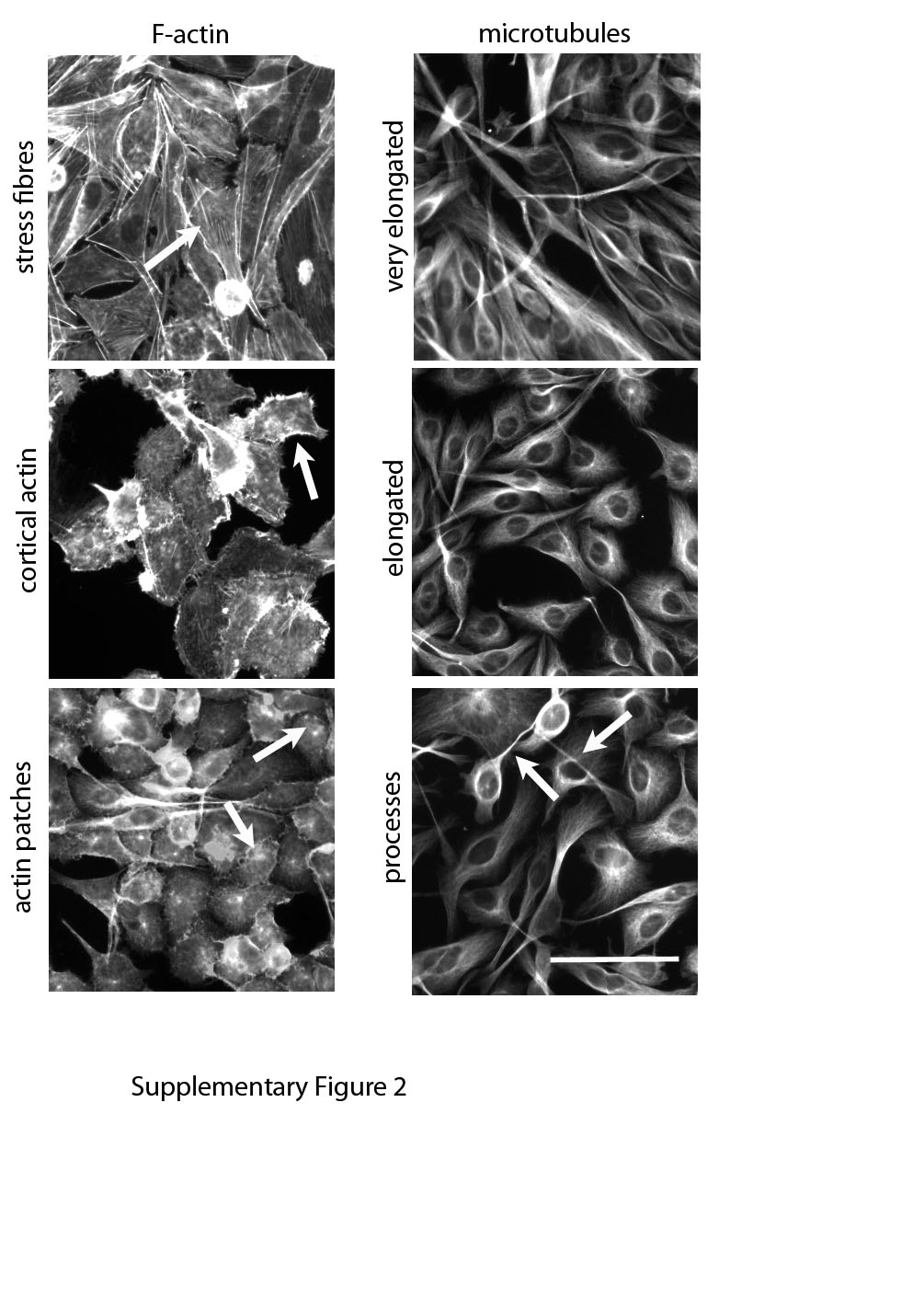

Supplement: Additional file 3 — Figure S2. Examples of actin and shape phenotypes in PMM-depleted cells. Description: Images show examples of cytoskeletal and shape phenotypes used to classify PMMs into groups in Table 2. PC3 cells were transfected with siRNA pools targeting ARC (stress fibres), FMNL1 (cortical actin), PDZK8 (actin patches), ZMYM4 (very elongated), FAM40B (elongated), or FMNL2 (processes). Images shown are taken from the images in Figure S1, and show the F-actin or microtubule channels separately as indicated. Arrows indicate examples of stress fibres, cortical actin, actin patches or processes. [file 1741-7007-9-54-S3.JPEG]

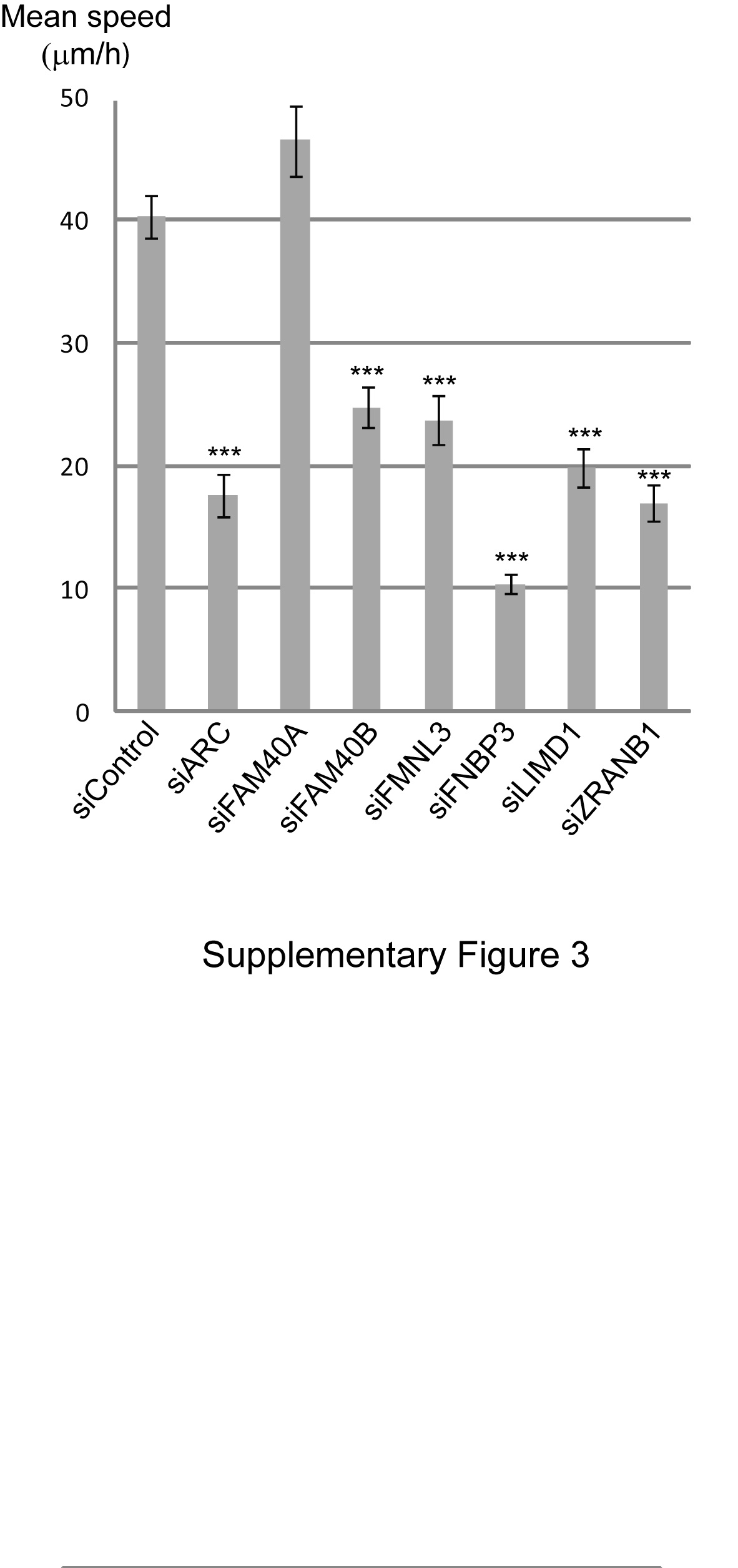

Supplement: Additional file 13 — Figure S3. Migration speeds of PMM-depleted PC3 cells. PC3 cells transfected with siRNAs for each of the indicated PMMs were imaged by time-lapse analysis for 14 h. Cells were tracked (Figure 3) and speeds determined. A total of 70 to 99 cells were tracked from three movies for each PMM. Values are means +/- s.e.m.; *** P ≤ 0.001, compared to control siRNA-transfected cells (unpaired Student's t-test). [file 1741-7007-9-54-S13.JPEG]
