# Supplementary material for: Identification and characterization of a set of conserved and new regulators of cytoskeletal organization, cell morphology and migration
Source: BMC Biol. 2011 Aug 11;9:54. doi: 10.1186/1741-7007-9-54 (PMC3201212; doi:10.1186/1741-7007-9-54)
Supplement: Additional file 4 — Table S2. Phenotypes induced by individual siRNAs. Description: Each of four different siRNA oligos targeting the indicated genes (listed in Table S2) was transfected into PC3 cells or HeLa cells. The number of oligos that gave the same morphological phenotype as the pool of four oligos (Figure 1 and Table S2 for PC3, Figure 6 for HeLa cells) is shown. NP, no phenotype; x, not tested (weak phenotype with pool). [file 1741-7007-9-54-S4.DOC]

|  | PC3 | HeLa |
| --- | --- | --- |
| ARC | **3**/4 | 4/4 |
| FAM40A | 4/4 | 4/4 |
| FAM40B | 3/4 | 3/4 |
| FMNL3 | 4/4 | NP |
| FNBP3 | 4/4 | 3/4 |
| LIMD1 | x | 4/4 |
| ZRANB1 | **3**/4 | 4/4 |

**Table S2. Phenotypes induced by individual siRNAs.** Each of 4 different siRNA oligos targeting the indicated genes (listed in Table S2) was transfected into PC3 cells or HeLa cells. The number of oligos that gave the same morphological phenotype as the pool of 4 oligos (Fig. 1 and Table 2 for PC3, Fig. 6 for HeLa cells) is shown. NP, no phenotype; x, not tested (weak phenotype with pool).
