# Supplementary material for: Identification and characterization of a set of conserved and new regulators of cytoskeletal organization, cell morphology and migration
Source: BMC Biol. 2011 Aug 11;9:54. doi: 10.1186/1741-7007-9-54 (PMC3201212; doi:10.1186/1741-7007-9-54)
Supplement: Additional file 14 — Table S3. siRNAs used for experiments. The names of the 26 human PMM genes and actin regulatory genes, and 4 siRNA sequences (sense strand) that were used as a pool for knockdown of each gene are shown. Only one siRNA was used for RhoA. [file 1741-7007-9-54-S14.DOC]

| **gene name** | **siRNA Sequence** |
| --- | --- |
| ARC | GUGAAGAACUGGGUGGAGU |
|  | AGGCAGACGGCUACGACUA |
|  | AGAAGUGGUGGGAGUUCAA |
|  | GGGUCAAGCGCGAGAUGCA |
| BRWD1 | GAAAUUGAGUGAUUGUGAA |
|  | CGAAAGAGAGUCUAUUUAA |
|  | GAGCUGUGUUUGACUGUAA |
|  | GACAGAUUCCGCUCUAUUA |
| BRWD3 | GACCUGAGACUAAUUAAUG |
|  | GGUGGUAUGUUCAUUACAA |
|  | GCAAGAAUUUGGCAGUAUC |
|  | GAUCGAAGCCGAGCUGUAU |
| C20ORF142 | GCAAAUACUUUCACAAGCG |
|  | GACGGUGGCUGACGAGAUC |
|  | GUACGGGACAUACGGGUUU |
|  | GCACGGCCAUCUGGUACAU |
| C21ORF2 | AAGCUAUGCUGCACACUGA |
|  | GGAUGAACGUGGCCUGAAG |
|  | GAAGAUGGUUCUGACCCGA |
|  | GGCAGCCGCCUCACAGAUA |
| CAMSAP1 | GAGGGAAGGUUAUAUGUUA |
|  | UCAAACAAGCCGAUUAUUC |
|  | UAUAAUAUUCGGCUUCUGA |
|  | GUGUUGAAGCCGAAUGUUA |
| CAPZB | GAAGUACGCUGAACGAGAU |
|  | GGAGUGAUCCUCAUAAAGA |
|  | GAGACAAGGUGGUGGGAAA |
|  | CACCAUGGAGUAACAAGUA |
| Cdc42 | GGAGAACCAUAUACUCUUG |
|  | GAUUACGACCGCUGAGUUA |
|  | GAUGACCCCUCUACUAUUG |
|  | CGGAAUAUGUACCGACUGU |
| CFL1 | UGACAGGGAUCAAGCAUGA |
|  | GCGGUGCUCUUCUGCCUGA |
|  | GUCAAGAUGCUGCCAGAUA |
|  | GCUAUGCCCUCUAUGAUGC |
| DIAPH1 | GAAGUGAACUGAUGCGUUU |
|  | GAAGAGAGAGCAACUCAUA |
|  | GGAGAUGGAUGACUUUAAU |
|  | GAUAUGAGAGUGCAACUAA |
| EPB41L4A | AGACACAGAUCUCGUUCGA |
|  | UCAAACACCAUCAGUAGGA |
|  | CGUAAACCUUGUGGAGAUA |
|  | GCGCUGUUCCGGAAGAAUU |
| FAM40A | GCAGCAAAUUUAUAGGUUA |
|  | GCAUGAAUGUUCUAAGACA |
|  | GCUGAUGACUCUCGAGAAG |
|  | UAGCGGACGUCUUGCCUGA |
| FAM40B | GAAGGCAACUCCUCACUAA |
|  | UAAAGCAGCACAAGUAUAU |
|  | GGGCCAACAUUGAGGCUUU |
|  | UGCCGGAGCUUACUACUGA |
| FMNL1 | AGGCGUACCUGGACAAUAU |
|  | GAGAAGGGGUUAAUCCGUA |
|  | GAAUUGGGCCCAGGAGUGA |
|  | GCCAAGCCAUUGAGGCGUA |
| FMNL2 | GAACCUACCUCCUGACAAA |
|  | UAAGAGAACUGGAAAUUUC |
|  | UAACAGACAUGUAUAUGAG |
|  | AAUUAGGCCUGGACGAAUA |
| FMNL3 | GCGAGGAGGUCACGAAAUC |
|  | UAAAGCUGCUGCGGCAAUA |
|  | UGUCAGCCAUUCGAAUUAA |
|  | CAGCGUCGAUGUCAUUUGG |
| FNBP3 | GGACAUAACUCUAGAAUCU |
|  | GGAAUGAUGUCGUCAGUAA |
|  | GGAAAGCCUUACUAUUAUA |
|  | GAUGCUGUCUGGGAAGAUA |
| HYPC | AGGCAAACCUUAUUACUAU |
|  | GGAAAGAGUACAAGUCGGA |
|  | CUACAAUGCUGACGACAAG |
|  | UCACACAGAUACCAGGAAU |
| LARP4 | CAUAAGCGUUGUAUUGUAA |
|  | UAGGAUGUCUGAUGUUGUU |
|  | CAAGGGCUAGUAAGGAUUA |
|  | GGACAGUUGAACAGAUAUA |
| LIMD1 | UCGAGGACCUGAACAUGUA |
|  | UCACUCAUGGAGACUAUUA |
|  | GCCGGAAGCUGAGAGGAAA |
|  | GAGUAGAGGCCCUGUCAAU |
| PDZK8 | AAACAGAGGAGUUCUAUUA |
|  | GGACUUACACUUCGUCUUG |
|  | GCACUACGCAAUCUGAGUA |
|  | GCUCAUGCCAAUCGGGUUA |
| PHIP | GAUGGGAGGUUGUUAGCUA |
|  | CGACAUGACAAUACAGUUA |
|  | CAACACAAUUAUCGUACAA |
|  | AUAUGGAGCUUAUACCUAA |
| Rac1 | AGACGGAGCUGUAGGUAAA |
|  | UAAGGAGAUUGGUGCUGUA |
|  | UAAAGACACGAUCGAGAAA |
|  | CGGCACCACUGUCCCAACA |
| RhoA | AUGGAAAGCAGGUAGAGUU |
| RhoU | GUACUGCUGUUUCGUAUGA |
|  | GAACGUCAGUGAGAAAUGG |
|  | CAGAGAAGAUGUCAAAGUC |
|  | AAGCAGGACUCCAGAUAAA |
| SH3D19 | GAACAAAGCCAAAAUAGUA |
|  | GAAGAUAGAUACAGAUUGG |
|  | GAACCUGGCUGAAGAAUCU |
|  | GACACCCUCUCUACAGUAA |
| SH3KBP1 | CAAGGUCAAUUGAAGUAGA |
|  | GGAGUGGACGCGUCAAAGA |
|  | CUAUCCAAGUCAAGUUUAA |
|  | ACGAGAGAUUAAACAGUUA |
| WAVE3 | CAUCGGACGUUACGGAUUA |
|  | GCUAACAACUUCUACAUCA |
|  | GGGCUGAAGUUCUAUACUG |
|  | GGCUGAAGUUCUAUACUGA |
| WTIP | GGACAUCUCAUCAUGGAAA |
|  | CAACGUGGGUGAGAAAGUG |
|  | GCGAGACUAUCACACGGUU |
|  | GCAGACGGCCGACAAAUGC |
| ZMYM3 | GUACCGGGCUCAACUAUUC |
|  | UAUCACAUGUGGAUCGUAA |
|  | GCCCAAUGGUGAACGAUAU |
|  | GGCCUCAUCUGACCUUUGU |
| ZMYM4 | GGAAGUACAUACAGUGGUG |
|  | GAAGAAGUCUAUAGUGGCU |
|  | GACACCAUGUUAACACGUA |
|  | GAAGAGCAUUUGUGGGAGU |
| ZMYM6 | UAAAGAAGAACCAGACAAU |
|  | GCAGUUGAACCCAGGCUUU |
|  | GAAAAUUGGUGGUGUGUCU |
|  | UAUCAUAAGACAGGAUCUA |
| ZNF135 | CGGAACAGCUCGGCACUUA |
|  | GCUCAGCACUUAUCGAACA |
|  | GGAGAAGCCAGACCUAAAU |
|  | CAACAGUGUCAUCUUGGUA |
| ZRANB1 | GAAGAAGAAUCUCCAAUUA |
|  | GAAAUAAACUGAACACUAG |
|  | CAGCAGAUAUUGAAGAUUU |
|  | UAAGUGGGCUUGUGAAUAU |

**Table S3.** **siRNAs used for experiments.** The names of the 26 human PMM genes and actin regulatory genes, and 4 siRNA sequences (sense strand) that were used as a pool for knockdown of each gene are shown. Only one siRNA was used for RhoA.
